# Global, regional, and national burden of syphilis, 1990–2021 and predictions by Bayesian age-period-cohort analysis: a systematic analysis for the global burden of disease study 2021

**DOI:** 10.3389/fmed.2024.1448841

**Published:** 2024-08-15

**Authors:** Wenxia Yu, Xiaoyan You, Wei Luo

**Affiliations:** Department of Clinical Laboratory, Qingdao Municipal Hospital, Qingdao, China

**Keywords:** syphilis, prevalence, age-standardized rate, disease burden, risk factor

## Abstract

**Objective:**

To comprehensively assess the global burden of syphilis and related risk factors over 1990–2021, forecast future disease trends, and understand the impact of syphilis on global health.

**Methods:**

Global Burden of Disease Study 2021 (GBD 2021) data were used for age-, sex-, and region-stratified analysis of the numbers and age-standardized rates (per 100,000 population) of syphilis incidence, prevalence, deaths, and disability-adjusted life years (DALYs). Next, a differential analysis of syphilis risk factors was performed. Finally, trends for years after 2021 were predicted using Bayesian age–period–cohort (BAPC) prediction models.

**Results:**

In 2021, the total number of syphilis prevalence globally was 70,541,482.80 (95% uncertainty interval: 54,910,897.66–88,207,651.97), with the highest numbers noted in Central Sub-Saharan Africa [4,622.60 (95% uncertainty interval: 3,591.97–5,753.45)]. Over 1990–2021, the global age-standardized prevalence and incidence rates increased, whereas the age-standardized death and DALY rates decreased. Among all groups, infants aged <5 years demonstrated the highest age-standardized DALY rates. Moreover, the lower the sociodemographic index (SDI), the higher was the age-standardized rate. The primary factor contributing to syphilis disease burden was identified to be unsafe sex. BAPC analysis revealed an overall increase in age-standardized prevalence rate in the <5-year age group over 1990–2035, and the highest age-standardized prevalence rate occurred in the 25–34-year age group.

**Conclusion:**

Between 1990 and 2021, syphilis occurrence and prevalence increased consistently. Projections indicated a continual increase in syphilis incidence in children aged <5 years, and age-standardized prevalence rates were the highest in adults aged 25–34 years. Our results regarding the epidemiological trends of syphilis and its variations across regions, age groups, and sexes may aid policymakers in addressing the global impact of the disease effectively.

## 1 Introduction

Syphilis is a chronic, multistage disease caused by *Treponema pallidum*, which can infect any organ in the human body and lead to diverse clinical outcomes (1). During the primary stage of the disease, chancres (i.e., primary lesions) typically form in the genital or anal area; they are usually painless, firm, and round and often go unnoticed (2). In the secondary stage, numerous symptoms primarily related to the skin may occur, which may include rashes often appearing on the palms of the hands and the soles of the feet, mucous membrane lesions, and generalized lymphadenopathy (2, 3). After a latency period lasting over decades, the disease may progress to the tertiary stage, affecting various organs. The tertiary stage may result in severe conditions including gummas (i.e., soft, tumor-like growths), cardiovascular syphilis (e.g., aortitis), and neurosyphilis (affecting the nervous system, potentially leading to paralysis, dementia, and sensory deficits) (2, 4).

Syphilis, which can be transmitted through sexual contact or vertically from mother to child, has a high prevalence in low-and middle-income countries (5). Globally, most syphilis cases occur in Sub-Saharan Africa, Southeast Asia, and South America, where congenital syphilis remains a major concern, accounting for up to 50% of all stillbirths (6). Syphilis prevalence is particularly high in Sub-Saharan Africa, where prenatal syphilis screening coverage is inadequate (7). Recent studies have indicated an increase in syphilis incidence, particularly among men who have sex with men (MSM) in high-and middle-income countries (5, 8). Moreover, syphilis incidence among women is increasing, which has led to an increase in congenital syphilis prevalence (9).

Syphilis is currently a major cause of adverse pregnancy outcomes in low-and middle-income countries (8). In developing countries, syphilis also contributes to hundreds of thousands of stillbirths and neonatal deaths annually (8). The World Health Organization (WHO) estimated 19.9 million cases of syphilis among people aged 15–49 years, with 6.3 million new cases, in 2016. The WHO has set a goal of a 90% reduction in global syphilis incidence by 2030 and a 50% reduction in congenital syphilis cases per 100,000 live births in 80% percent of affected countries (1, 10).

The occurrence of syphilis varies across countries and regions, with most new cases occurring in low-and middle-income countries. Among these, pregnant women in Africa bear the highest burden (8). In the United States, 2021 data revealed a significant increase in the number of syphilis cases, with more than 171,000 reported cases across all stages, indicating a 68% increase in prevalence compared with that in 2017. Moreover, a 28% increase occurred in syphilis case numbers from 2020 to 2021, highlighting the persistent public health challenge posed by syphilis, even during the COVID-19 pandemic (9).

Analysis of up-to-date statistics is essential for effective prevention, control, and treatment of infectious diseases, such as syphilis. The Global Burden of Diseases, Injuries, and Risk Factors Study 2021 (GBD 2021), a comprehensive epidemiological study conducted globally, assessed mortality and years of life lost (YLLs) across 288 causes of death, stratified by age, sex, location, and year, in 204 countries and territories, as well as 811 subnational regions, over 1990–2021 (11). In the present study, we used GBD 2021 data to retrospectively analyze the disease burden of syphilis worldwide to provide the most current estimates for ongoing epidemiological research on syphilis.

## 2 Methods

### 2.1 Data sources

We retrospectively analyzed GBD 2021 data, available at https://vizhub.healthdata.org/gbd-results/. GBD 2021 analyzed disease and injury burden by estimating years lived with disability (YLDs), YLLs, disability-adjusted life years (DALYs), and healthy life expectancy (HALE) for 371 diseases and injuries using 100,983 data sources (12). The data sources included various platforms, including vital registration systems, verbal autopsies, censuses, household surveys, disease-specific registries, and health service contact data (12). DALYs were calculated as the sum of YLLs and YLDs (13).

In the current study, we also analyzed the burden across different sociodemographic index (SDI) quintiles. SDI is a composite measure representing the geometric mean of lag-distributed income *per capita*, average years of schooling, and fertility rate among females aged <25 years in a specific location. SDI scores range from 0 to 100, with 0 indicating the lowest income, fewest years of schooling, and highest fertility and 100 indicating the highest income, most years of schooling, and lowest fertility (12).

### 2.2 Statistical analysis

Estimation methods used in GBD 2021 have been summarized previously (11, 12, 14). GBD 2021 used the Bayesian DisMod-MR 2.1 model, which provided a 95% uncertainty interval (95% UI) for each estimate.

In the current study, all statistical analyses and visualizations were conducted using R version 4.2.1 [R Core Team (2021)]. We employed incidence, prevalence, deaths, DALYs, and age-standardized rates (per 100,000 population) to describe the global disease burden of syphilis, stratified by regions, sexes, and age groups. Moreover, Bayesian age–period–cohort (BAPC) prediction models were used to estimate future syphilis prevalence. Finally, the major risk factors for syphilis over 1990–2021 were analyzed.

## 3 Results

### 3.1 Global burden of syphilis

**Figure 1:**
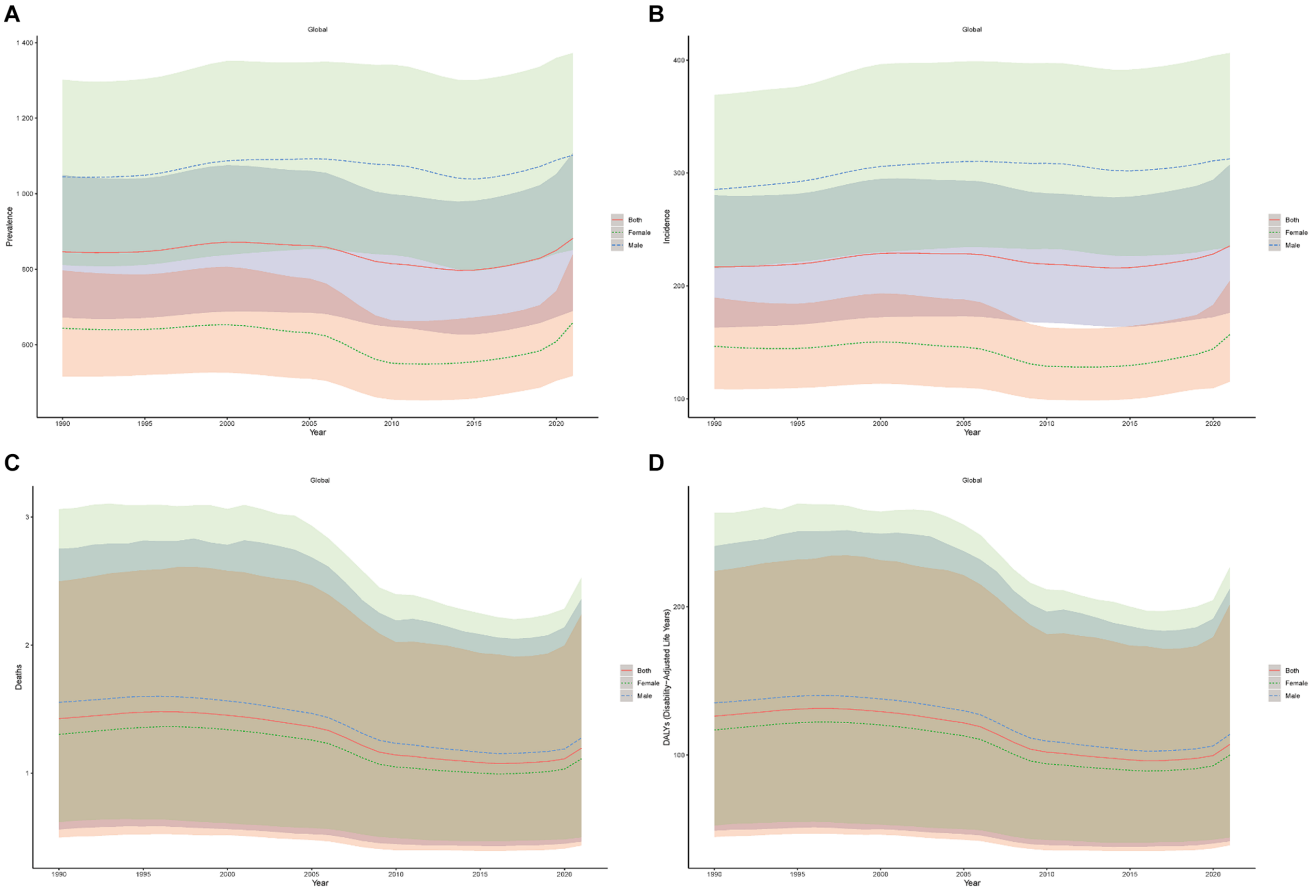
Age-standardized syphilis prevalence **(A)**, incidence **(B)**, death **(C)**, and DALY **(D)** rates (per 100,000 population) over 1990–2021.

From 1990 to 2021, the global disease burden of syphilis increased significantly in terms of age-standardized prevalence and incidence rates (Figures 1A,B). In contrast, age-standardized death and DALY rates decreased (Figures 1C,D). In 2021, age-standardized DALY rates for syphilis were lower in female individuals than in male individuals (Figures 1A–D).

Syphilis prevalence numbers increased from 45,459,660.64 (95% UI: 36,008,443.28–56,513,622.10) in 1990 to 70,541,482.80 (95% UI: 54,910,897.66–88,207,651.97) in 2021, representing an increase of 25,081,822.16 (95% UI: 18,902,454.38–31,694,029.875). Furthermore, age-standardized prevalence rates increased from 846.29 (95% UI: 672.18–1,049.61) in 1990 to 881.60 (95% UI: 689.36–1,108.11) in 2021, indicating an increase of 35.31 (95% UI: 17.18–58.50; Table 1). Syphilis incidence numbers increased from 11,974,753.64 (95% UI: 8,932,531.75–1,555,536.31) in 1990 to 18,696,009.14 (95% UI: 114,033,725.79–24,331,643.87) in 2021, demonstrating in an increase of 6,721,255.50 (95% UI: 5,101,194.04–8,776,274.56). Moreover, age-standardized incidence rates increased from 216.93 (95% UI: 163.26–280.24) in 1990 to 235.47 (95% UI: 176.40–307.43) in 2021, indicating an increase of 18.54 (95% UI: 13.14–27.19; Table 1).

**Table 1:** Numbers and age-standardized rate (per 100,000 population) of syphilis prevalence, incidence, deaths, and DALYs in 2021, stratified by SDI and global disease burden in each region over 1990–2021.

Region	Prevalence		Incidence		Deaths		DALYs	
	Number (95% UI)	Age-standardized rate (95% UI)	Number (95% UI)	Age-standardized rate (95% UI)	Number (95% UI)	Age-standardized rate (95% UI)	Number (95% UI)	Age-standardized rate (95% UI)
High SDI	3686909.88 (2869224.05–4648196.14)	329.91 (252.24–417.53)	1000727.41 (760621.79–1306083.44)	94.64 (70.56–123.45)	373.95 (242.12–595.66)	0.05 (0.02–0.09)	25837.57 (14325.53–46514.58)	4.07 (1.76–8.20)
High-middle SDI	6015578.19 (4605534.89–7624360.83)	443.13 (338.52–558.87)	1580600.78 (1199673.81–2052697.51)	122.16 (90.59–159.87)	1621.42 (722.50–3162.68)	0.26 (0.11–0.53)	141939.57 (60265.40–280930.85)	23.60 (9.37–47.93)
Middle SDI	18958159.20 (14654369.84–23867714.37)	740.81 (575.43–938.15)	4880941.38 (3650324.44–6407225.48)	193.33 (143.36–253.00)	10676.46 (4272.92–21176.22)	0.68 (0.26–1.36)	953820.18 (374561.88–1896954.53)	60.76 (23.27–121.90)
Low-middle SDI	21202039.35 (16576507.46–26708242.33)	1064.8 (833.48–1321.88)	5556832.71 (4106335.57–7294019.56)	271.96 (202.72–354.69)	23800.81 (9289.67–47743.44)	1.29 (0.51–2.57)	2129082.67 (838152.98–4290176.37)	114.24 (45.04–229.95)
Low SDI	20633485.86 (16240477.79–26080390.35)	1988.15 (1566.98–2480.47)	5665495.41 (4266423.36–7356971.15)	519.69 (3995.12–667.48)	38156.7 (14874.41–76345.73)	2.30 (0.92–4.59)	3433884.02 (1342531.55–6877866.09)	203.37 (80.86–403.18)
Oceania	171830.62 (136017.88–216598.51)	1210.07 (950.15–1511.73)	37271.76 (27932.95–48629.15)	253.51 (192.03–329.96)	569.86 (208.05–1111.27)	2.81 (1.04–5.49)	51553.77 (19084.49–100196.74)	253.90 (95.02–491.41)
North Africa and Middle East	2239177.43 (1703555.72–2818334.48)	342.77 (262.15–427.51)	556086.61 (410662.19–730852.70)	84.35 (62.01–110.56)	2901.07 (1105.61–6087.64)	0.51 (0.20–1.06)	259042.60 (98108.09–545280.69)	45.07 (17.11–94.81)
Eastern Sub-Saharan Africa	10922130.12 (8625945.74–13716388.39)	2794.83 (2222.66–3495.64)	2998005.99 (2251473.61–3873571.03)	725.34 (552.38–926.90)	20198.54 (8108.76–40520.94)	3.20 (1.31–6.35)	1818902.44 (724092.79–3655044.90)	283.39 (116.36–563.39)
Southeast Asia	6344517.62 (4943307.00–7898830.10)	856.96 (672.67–1065.74)	1581322.50 (1184607.28–2072767.58)	212.64 (159.40–277.53)	4728.01 (1723.90–9581.15)	0.87 (0.32–1.77)	430983.32 (159032.26–866147.28)	78.98 (28.75–159.52)
Central Europe	184103.50 (141927.50–235751.70)	160.74 (121.83–203.00)	50752.83 (38353.17–66127.51)	47.27 (34.83–61.78)	17.87 (12.11–27.02)	0.02 (0.01–0.04)	1169.30 (698.89–2013.11)	1.61 (0.76–3.19)
Western Sub-Saharan Africa	7055169.90 (5486954.83–8890200.89)	1564.47 (1214.35–1954.25)	1880943.83 (1389428.96–2448718.02)	400.61 (299.51–518.76)	14007.91 (5273.99–28107.40)	1.74 (0.69–3.42)	1258845.69 (473654.35–2516067.99)	152.59 (59.75–302.96)
Central Asia	219388.28 (167528.65–275589.38)	218.38 (167.51–273.34)	62080.00 (46967.99–81144.66)	62.51 (47.21–81.90)	86.99 (42.32–168.58)	0.09 (0.05–0.17)	7268.91 (3412.93–14040.13)	7.44 (3.53–14.31)
Eastern Europe	411366.37 (321942.14–522057.71)	189.58 (145.25–239.57)	108392.51 (83765.67–140554.06)	53.91 (40.30–70.54)	56.37 (49.20–68.72)	0.02 (0.02–0.02)	3620.04 (2963.12–4395.43)	1.45 (1.22–1.71)
Australasia	92107.59 (70049.19–117300.33)	290.66 (218.02–367.52)	26184.61 (19858.13–33996.52)	86.61 (64.89–113.11)	5.84 (4.67–7.42)	0.01 (0.01–0.01)	171.44 (139.42–210.36)	0.42 (0.34–0.53)
Southern Latin America	475830.50 (367506.30–599294.55)	675.97 (523.67–853.01)	129998.67 (96560.35–168866.03)	186.81 (139.23–243.29)	46.10 (35.72–56.73)	0.09 (0.07–0.12)	3206.3 (2299.11–4039.66)	7.29 (4.91–9.37)
Caribbean	397147.04 (316227.54–494822.74)	817.66 (651.40–1019.36)	99756.58 (76220.04–128186.90)	205.67 (156.72–263.81)	687.84 (272.55–1387.44)	1.78 (0.69–3.59)	60802.82 (23610.12–121357.05)	157.86 (60.23–316.41)
Southern Sub-Saharan Africa	2176239.09 (1707114.96–2750488.90)	2549.28 (2025.73–3213.08)	538684.64 (398503.72–704709.33)	623.40 (467.28–812.80)	2231.64 (875.14–4647.93)	2.87 (1.13–5.96)	200273.51 (78649.67–416844.88)	256.15 (100.69–533.06)
Western Europe	1021101.66 (797492.35–1285358.38)	237.98 (179.98–298.15)	288582.77 (216633.38–372135.61)	71.20 (52.69–92.95)	115.58 (79.77–173.62)	0.03 (0.02–0.06)	6790.74 (3856.57–12023.01)	2.57 (1.08–5.08)
Central Sub-Saharan Africa	5697404.46 (4413535.96–7260370.39)	4622.60 (3591.97–5753.45)	1663015.46 (1242841.11–2164179.92)	1266.92 (956.83–1630.00)	7733.51 (2969.09–15158.01)	3.74 (1.45–7.24)	697894.52 (267611.48–1368189.39)	332.9 (131.12–647.20)
Tropical Latin America	1894677.95 (1576942.79–2295588.66)	803.23 (672.41–971.54)	453161.67 (344831.55–569831.28)	192.44 (1946.94–241.35)	400.66 (301.00–569.14)	0.22 (0.16–0.32)	31780.98 (22968.48–46569.49)	18.12 (12.83–27.05)
High-income North America	1399924.99 (1094296.29–1757728.10)	374.07 (285.88–473.52)	378735.15 (286995.57–490376.02)	106.42 (79.20–138.42)	63.76 (55.38–73.32)	0.01 (0.01–0.01)	3804.81 (3060.15–4788.36)	0.78 (0.64–0.96)
High-income Asia Pacific	610077.17 (473808.24–780787.00)	334.60 (257.56–423.96)	165213.51 (123337.07–213757.34)	97.96 (72.36–128.01)	78.17 (52.16–124.02)	0.07 (0.03–0.15)	4655.99 (2500.54–8768.73)	6.16 (2.51–13.33)
Global	70541482.80 (54910897.66–88207651.97)	881.60 (689.36–1108.11)	18696009.14 (14033725.79–24331643.87)	235.47 (176.40–307.43)	74705.62 (29410.04–146947.03)	1.19 (0.47–2.36)	6691400.44 (2637154.19–13200357.40)	107.13 (41.77–212.11)
Central Latin America	1215442.51 (923911.36–1539691.32)	455.81 (347.86–575.51)	335511.20 (249535.82–442075.02)	125.20 (93.36–164.44)	154.49 (116.67–219.30)	0.07 (0.05–0.11)	10712.43 (7640.58–16326.00)	5.15 (3.56–8.11)
Andean Latin America	578679.51 (440685.80–733703.80)	830.29 (637.88–1043.38)	165006.64 (123805.94–212904.81)	234.14 (176.29–300.45)	630.35 (230.52–1335.53)	1.06 (0.39–2.24)	56293.78 (20460.11–119498.50)	94.18 (34.14–200.02)
South Asia	19270979.29 (14907736.82–24423122.93)	984.62 (767.45–1237.76)	5059818.31 (3711516.49–6662314.26)	251.82 (187.19–328.48)	17954.34 (7084.31–37719.93)	1.19 (0.48–2.50)	1602966.11 (621649.15–3386673.25)	105.39 (40.66–223.07)
East Asia	8164187.20 (6176234.55–10529902.02)	530.47 (400.52–681.98)	2117483.88 (1593933.09–2770057.12)	145.97 (106.55–192.75)	2036.75 (825.28–4370.17)	0.35 (0.13–0.76)	180660.93 (71520.69–390766.83)	30.91 (11.55–67.99)

Syphilis deaths numbers decreased from 89,416.20 (95% UI: 34,514.83–172,155.23) in 1990 to 74,705.62 (95% UI: 29,410.04–146,947.03) in 2021, demonstrating a decrease of 14,710.58 (95% UI: 5,104.79–25,208.20). Age-standardized death rates also decreased from 1.42 (95% UI: 0.56–2.75) in 1990 to 1.19 (95% UI: 0.47–2.36) in 2021, indicating a decrease of 0.23 (95% UI: 0.09–0.39; Table 1). Syphilis DALY numbers increased from 89,416.20 (95% UI: 34,514.83–172,155.23) in 1990 to 74,705.62 (95% UI: 29,410.04–146,947.03) in 2021, resulting in a decrease of 14,710.58 (95% UI: 5,104.79–25,208.20). Age-standardized DALY rates also decreased from 126.18 (95% UI: 48.91–240.98) in 1990 to 107.13 (95% UI: 41.76–212.11) in 2021, indicating a decrease of 19.05 (95% UI: 7.15–28.87; Table 1).

### 3.2 Regional burden of syphilis

In 2021, the age-standardized syphilis incidence rate (per 100,000 population) was the highest in Central Sub-Saharan Africa [1,266.92 (95% UI: 956.83–1,630.00)], followed by Eastern Sub-Saharan Africa region [725.34 (95% UI: 552.38–926.90)] and Southern Sub-Saharan Africa [623.40 (95% UI: 467.28–812.80)]. In contrast, it was the lowest in Central Europe [47.27 (95% UI: 34.83–61.78)], preceded by Eastern Europe [53.91 (95% UI: 40.30–70.54)] and Central Asia [62.51 (95% UI: 47.21–81.90)]. The age-standardized syphilis prevalence rate was the highest in Central Sub-Saharan Africa [4,622.60 (95% UI: 3,591.97–5,753.45)], followed by Eastern Sub-Saharan Africa [2,794.83 (95% UI:2,222.66–3,495.64)] and Southern Sub-Saharan Africa [2,549.28 (95% UI: 2,025.73–3,213.08)]. In contrast, it was the lowest Central Europe region [160.74 (95% UI: 121.83–203.00)], preceded by Eastern Europe [189.58 (95% UI: 145.24–239.57)] and Central Asia [218.38 (95% UI: 167.51–273.34)]. The age-standardized syphilis death rate was the highest in Central Sub-Saharan Africa [3.74 (95% UI: 1.45–7.24)], followed by Eastern Sub-Saharan Africa [3.20 (95% UI: 1.31–6.35)] and Southern Sub-Saharan Africa [2.87 (95% UI:1.13–5.96)]. In contrast, it was the lowest in high-income North America [0.01 (95% UI: 0.01–0.01)] and Australasia [0.01 (95% UI: 0.01–0.01)], preceded by Central Europe [0.02 (95% UI: 0.01–0.04); Table 1]. The age-standardized syphilis DALY rate was the highest in Central Sub-Saharan Africa [332.90 (95% UI: 131.12–647.20)], followed by Eastern Sub-Saharan Africa [283.39 (95% UI: 116.36–563.39)] and Southern Sub-Saharan Africa [256.15 (95% UI: 100.69–533.06)]. In contrast, it was the lowest in Australasia [0.42 (95% UI: 0.34–0.53)], preceded by high-income North America [0.78 (95% UI: 0.64–0.96)] and Eastern Europe [1.45 (95% UI: 1.22–1.71); Table 1].

**Figure 2:**
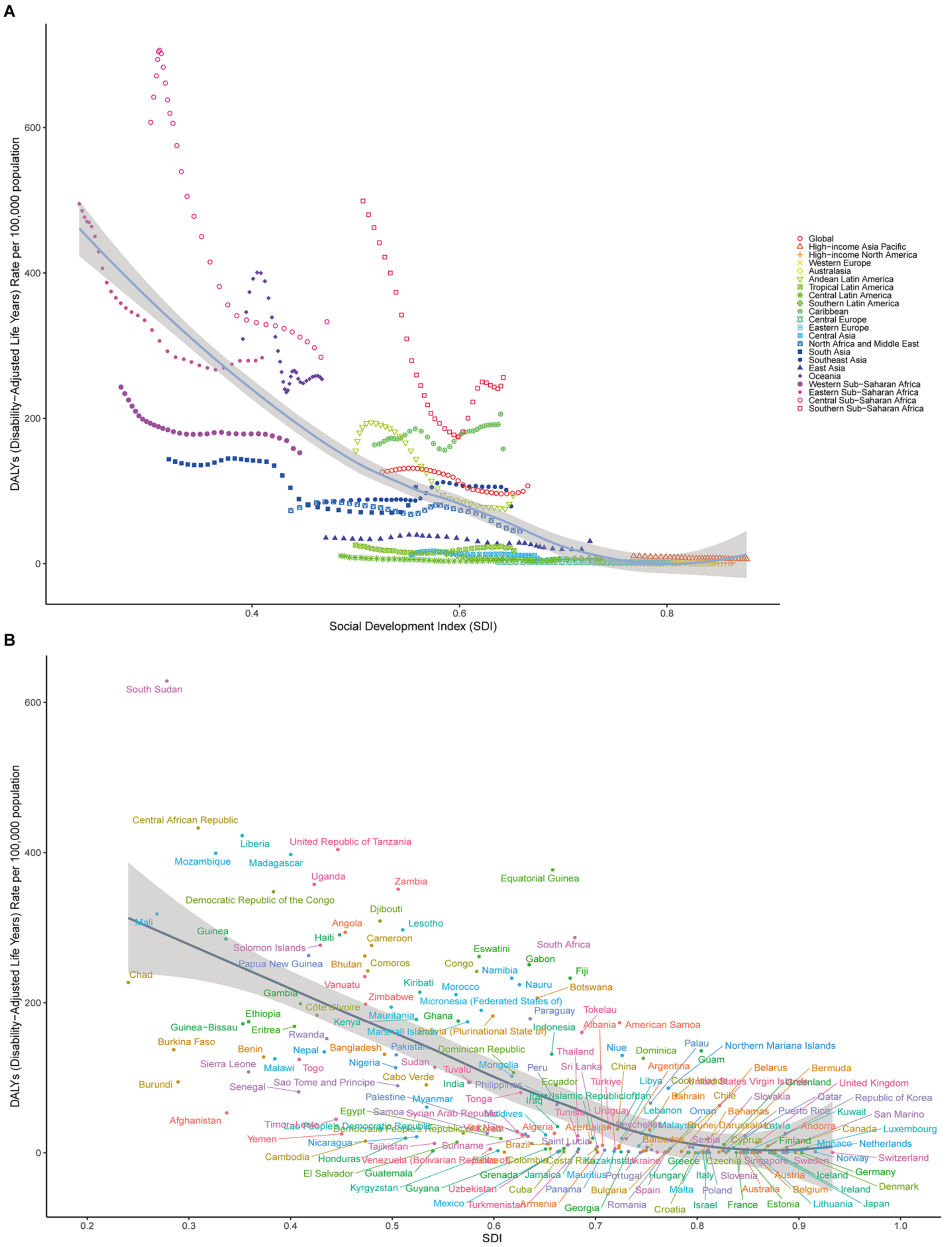
**(A)** Age-standardized syphilis DALY rates globally and in 21 disease-burden regions stratified by SDI over 1990–2021. The expected age-standardized rates in 2021, based solely on SDI, are represented by the black line. For each region, points from left to right depict estimates for each year from 1990 to 2021. **(B)** Age-standardized syphilis DALY rates in 204 countries by SDI in 2021. The expected age-standardized rates in 2021, based solely on SDI, are represented by the black line.

Figures 2A,B compares the observed global age-standardized DALY rates and the expected SDI in each region. Over 1990–2021, Central Sub-Saharan Africa and Southern Sub-Saharan Africa demonstrated significant changes. Moreover, Oceania, the Caribbean, Central Sub-Saharan Africa, and Southern Sub-Saharan Africa had higher than expected SDI levels (Figure 2A). Most medium SDI regions demonstrated varied patterns over 2009–2021, with some regions remaining well below the expected levels, whereas others remaining well above the expected levels.

### 3.3 National burden of syphilis

**Figure 3:**
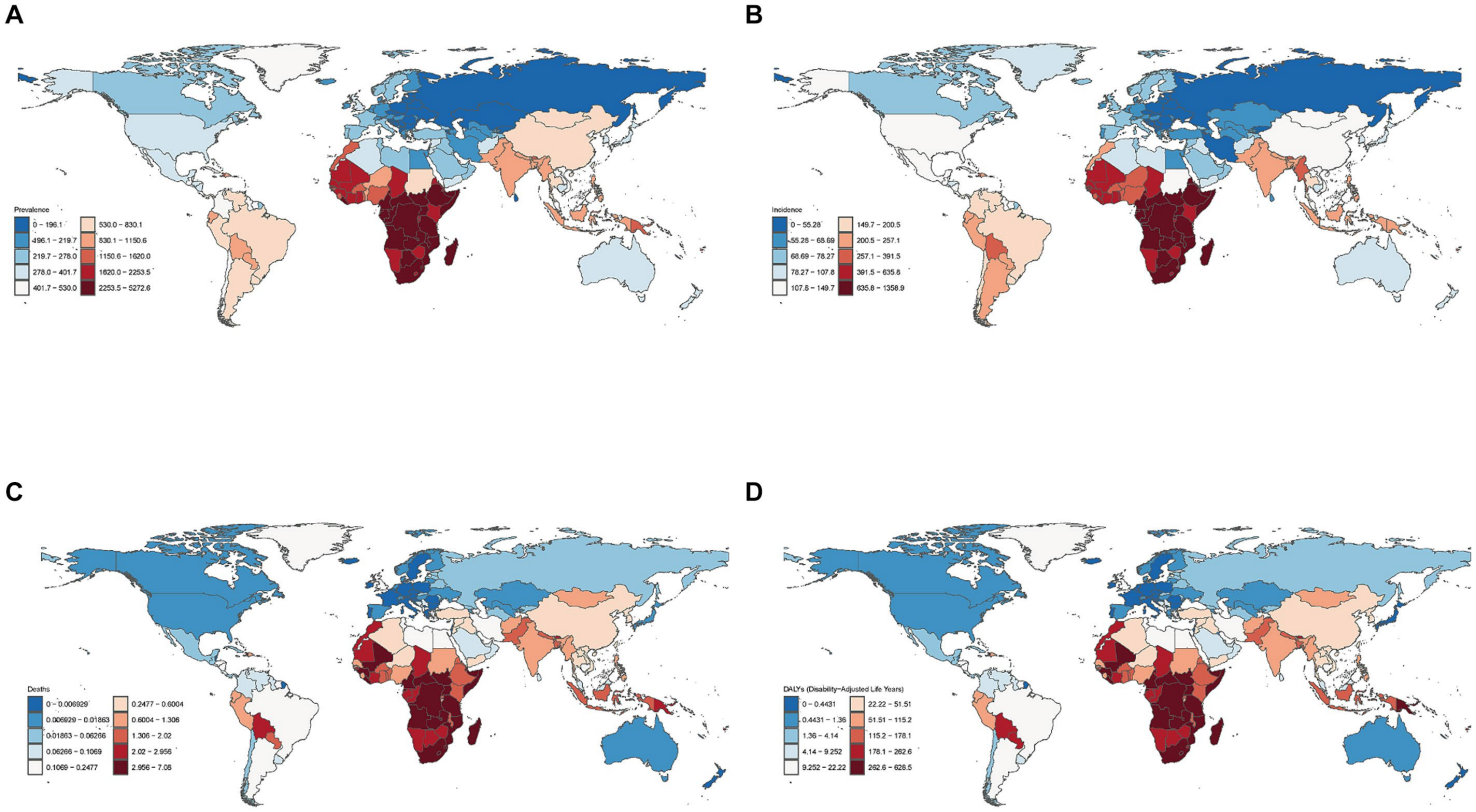
Global age-standardized prevalence **(A)**, incidence **(B)**, death **(C)**, and DALY **(D)** rates (per 100,000 population) in 2021 in 204 countries and territories.

In 2021, the following three countries demonstrated the highest age-standardized syphilis prevalence rates: Central African Republic [5,272.60 (95% UI: 4,211.16–6,584.16)], Equatorial Guinea [4,959.14 (95% UI: 3,816.33–6,196.37)], and Democratic Republic of the Congo [4,677.81 (95% UI: 3,635.61–5,795.34)]. In contrast, Slovenia [143.92 (95% UI: 107.09–185.74)], Slovakia [139.62 (95% UI:105.27–175.79)], and Bulgaria [129.42 (95% UI: 96.44–166.53)] exhibited the lowest age-standardized syphilis prevalence rates (Figure 3A; Supplementary Table S1).

In terms of age-standardized syphilis incidence rates, the top three countries were Equatorial Guinea [1358.92 (95% UI: 1,025.63–1,773.72)], Central African Republic [1,319.54 (95% UI: 1,008.39–1,701.85)], and Democratic Republic of the Congo [1,282.99 (95% UI: 964.13–1,658.13)]. In contrast, Croatia [44.96 (95% UI:33.66–58.70)], Slovakia [942.52 (95% UI: 31.77–55.09)], and Bulgaria [41.00 (95% UI: 30.59–53.14)] exhibited the lowest age-standardized syphilis incidence rates (Figure 3B; Supplementary Table S1).

In terms of age-standardized syphilis death rates, the top three countries were South Sudan [7.08 (95% UI: 2.76–14.61)], the Central African Republic [4.86 (95% UI: 1.97–9.53)], and Liberia [4.70 (95% UI:1.82–9.61)]. In contrast, Poland [0.0019 (95% UI: 0.0015–0.0026)] demonstrated the lowest age-standardized syphilis death rates, preceded by Malta [0.0023 (95% UI: 0.0018–0.0029)] and Sweden [0.0032 (95% UI: 0.0026–0.0040)] (Figure 3C; Supplementary Table S1).

Finally, in terms of age-standardized syphilis DALY rates, the top three countries were South Sudan [628.48 (95% UI: 240.55–1,292.40)], the Central African Republic [432.80 (95% UI:175.39–847.94)] and Liberia [422.51 (95% UI: 160.30–864.57)]. In contrast, Sweden [0.22 (95% UI: 0.16–0.33)] had the lowest age-standardized syphilis DALY rates, preceded by Malta [0.13 (95% UI: 0.09–0.21)] and Croatia [0.19 (95% UI: 0.14–0.27); Figure 3D; Supplementary Table S1].

### 3.4 Age-specific burden of syphilis

**Figure 4:**
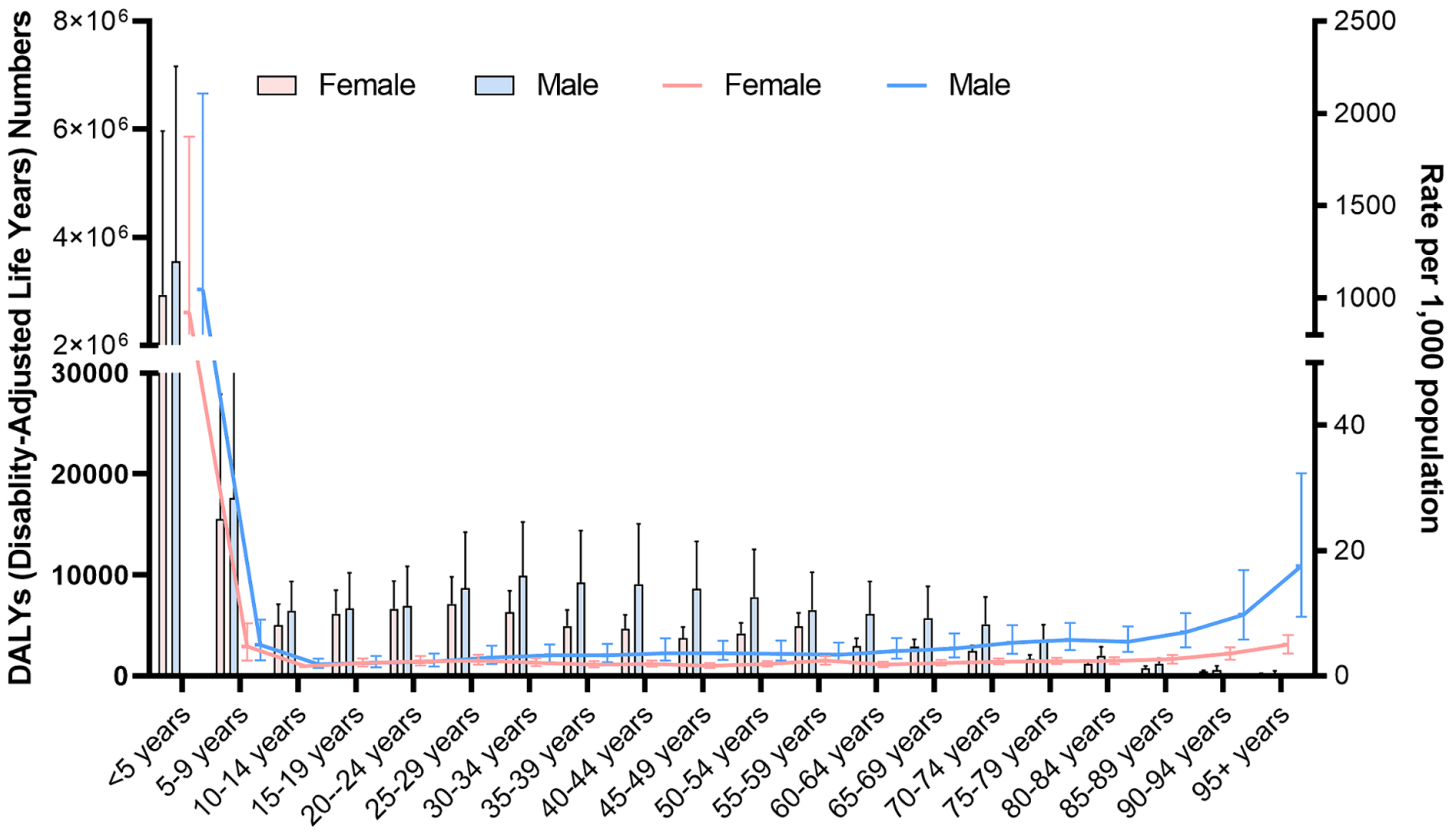
Age-standardized DALY rates (per 100,000 population) stratified by sex for different age groups in 2021.

Among all age groups, infants aged <5 years demonstrated the highest age-standardized DALY rates [985.70 (95% UI: 370.99–1973.98)]. In particular, female and male infants aged <5 years exhibited age-standardized DALY rates of 921.72 (95% UI: 350.72–1874.78) and 1045.58 (95% UI: 389.39–2108.05), respectively (Figure 4).

### 3.5 Risk factors

**Figure 5:**
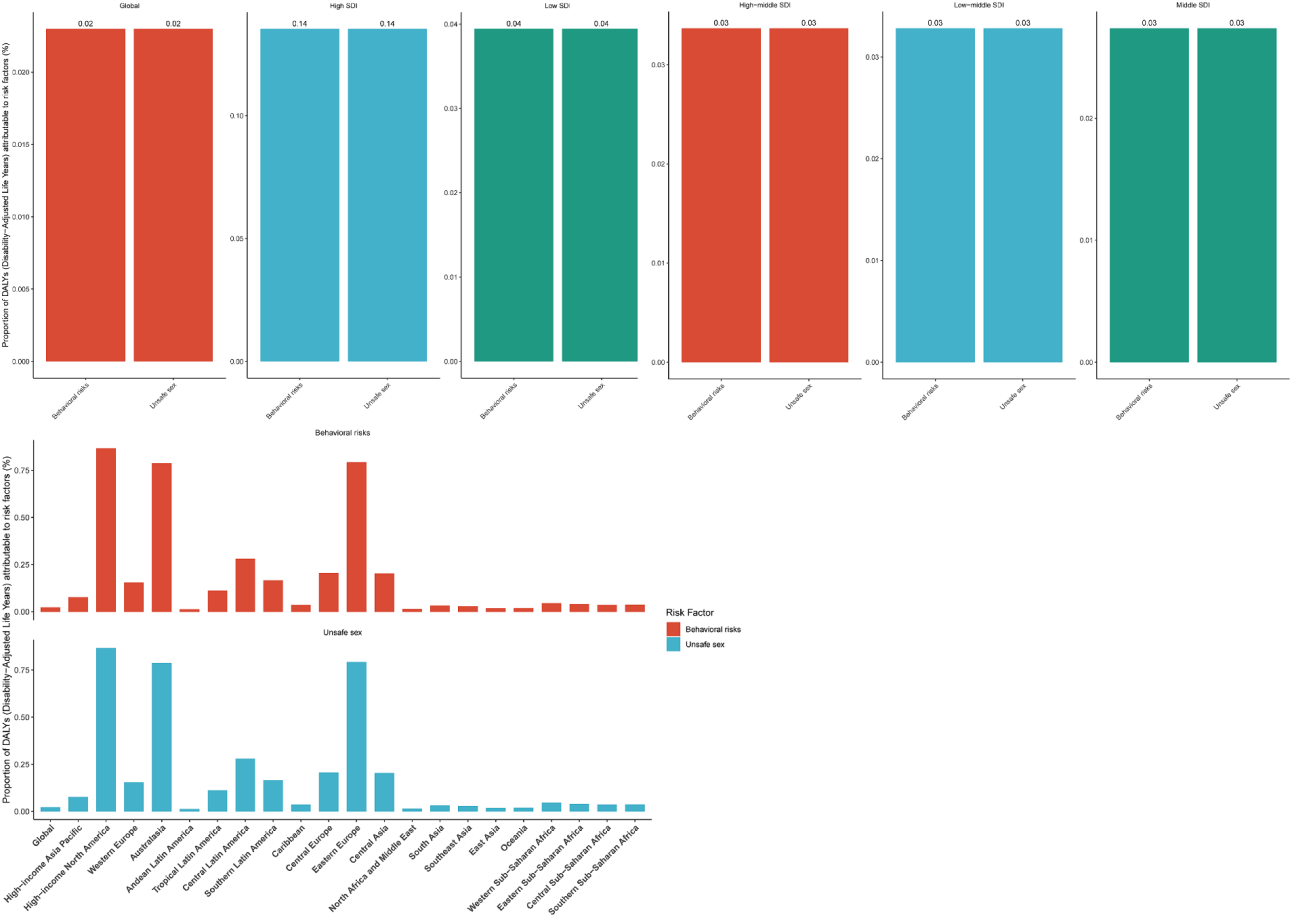
Percentages of age-standardized DALY rates (per 100,000 population) attributable to different regions in 2021 for various risk factors.

In 2021, unsafe sex was the syphilis risk factor attributable to the largest population of all ages (Figure 5). The percentage of syphilis cases attributable to unsafe sex varied by region, with high SDI areas demonstrating significantly higher rates than other areas. Moreover, among the 21 disease-burden regions, high-income North America, Australasia, and Eastern Europe demonstrated the highest contribution rates.

### 3.6 BAPC analysis

**Figure 6:**
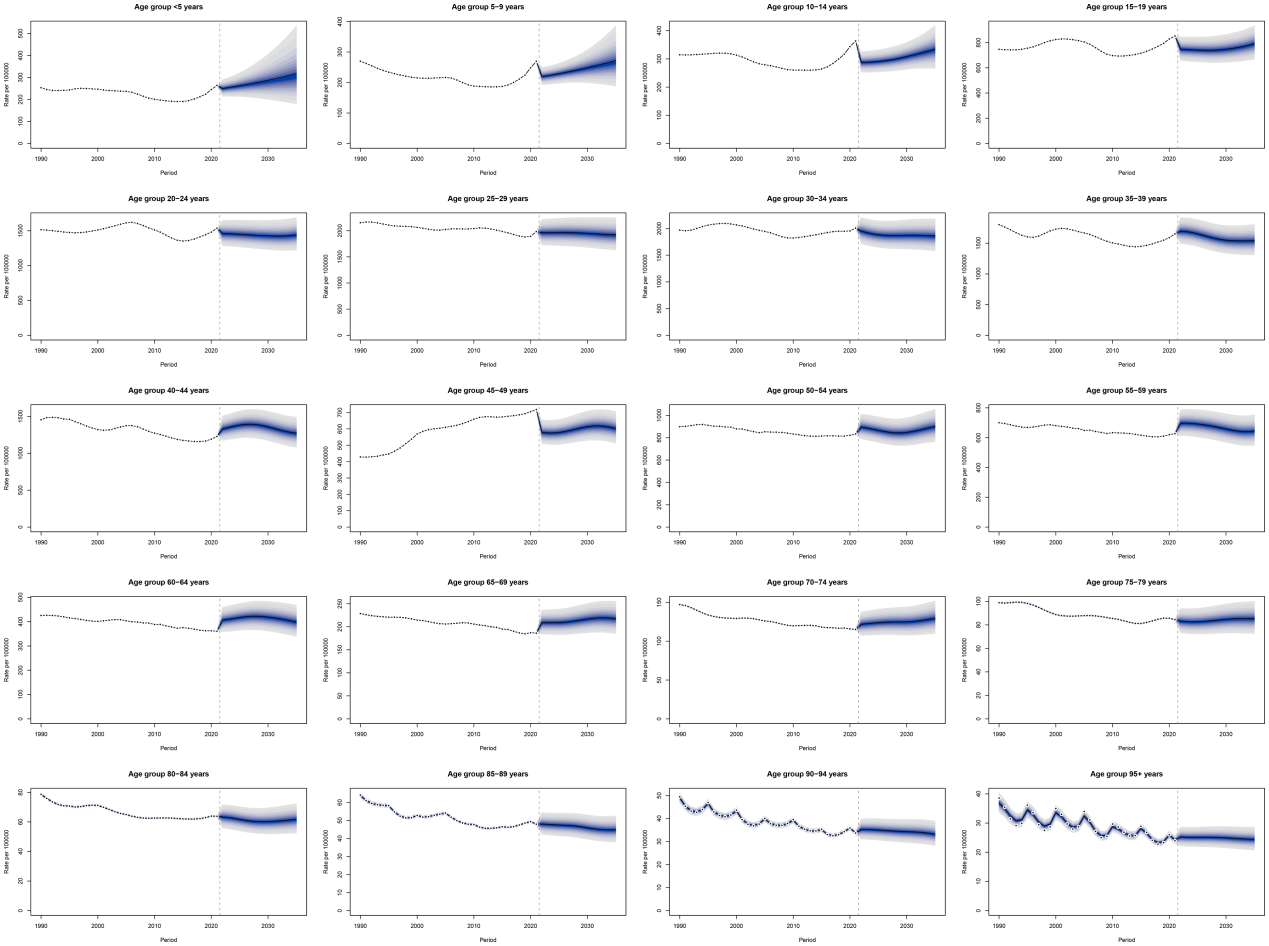
Trends in age-standardized prevalence rates from 2019 to 2035, predicted using BAPC prediction models.

BAPC prediction models indicated an overall increase in age-standardized syphilis prevalence rates from 1990 to 2035 for the <5-year age group, with an initial decline followed by an increase for the 5–19-year age group. The highest age-standardized syphilis prevalence rates occurred in the 25–34-year age group. The forecast results demonstrated a relatively stable trend of age-standardized prevalence rates from 2021 to 2035 for the 20–29-and 75–79-year age groups; however, a smaller decline was noted for the 20–39-and > 80-year age groups. The 40–44-and 55–59-year age groups demonstrated an upward trend, followed by a gradual downward trend. The 45–49-year age group demonstrated a decrease at first, followed by a gradual increase. The 65–74-year age group demonstrated a gradual increase. The 50–54-year age group demonstrated the trend of an initial increase, followed by a decline and then an increase again. Finally, the 60–64-year age group demonstrated an initial increase, followed by a decrease (Figure 6).

## 4 Discussion

In this study, we utilized publicly available modeling data and methods from GBD 2021 to provide the most up-to-date, comprehensive information regarding syphilis incidence, prevalence, death, and DALY rates in 204 countries and territories over 1990–2021. Our results facilitated estimation of the impact of syphilis on global health. The GBD 2021 results demonstrated an increase in global age-standardized incidence and prevalence rates, largely attributable to advancements in treatment strategies for syphilis. In particular, penicillin has remained the primary treatment modality for syphilis since 1943, making the disease effectively treatable (15).

The GBD 2021 results demonstrated that in 2021, the age-standardized DALY rates were higher in male individuals than in female individuals, possibly because of an increase in the MSM population (16). Several scholars have examined the relationship between MSM and syphilis development in different regions (17, 18). Globally, MSM may account for a large proportion of syphilis cases (19). In the United States, the prevalence of primary and secondary syphilis is highest among MSM, particularly those younger and from minority groups (20). Similarly, in Western Europe, MSM constitute the majority of primary and secondary syphilis cases and represent the group with the highest syphilis risk (19). Since 2000, the case numbers of MSM with syphilis have increased significantly in most Western countries (21). To address this issue, implementation of multiple strategies, including strengthening and targeting current syphilis screening and detection programs and providing timely treatment for syphilis cases, is highly warranted. The global distribution of syphilis varies significantly, with the burden being predominant in African regions. The reasons for these differences may be closely related to factors such as socioeconomic status, educational level, extent of sexual health knowledge dissemination, and unequal medical resource distribution (22).

Our findings revealed that infants aged <5 years had the highest age-standardized DALY rates among all age groups. Recent research has demonstrated a notable increase in the number of syphilis cases among women of childbearing age since 2013, consequently leading to an increase in congenital syphilis prevalence (23, 24). According to WHO data, congenital syphilis prevalence was 0.69%, with 473 cases per 100,000 live births, in 2016. Notably, syphilis is the second leading cause of stillbirths, after malaria (16). Most syphilis infections occur in newborns and infants due to fetal infection resulting from maternal spirochetemia. Congenital syphilis significantly contributes to fetal and neonatal mortality worldwide, leading to stillbirths, miscarriages, preterm births, birth defects, and lifelong physical or neurological impairments. The only recommended treatment for syphilis during pregnancy is benzathine penicillin G use (25, 26). The increase in the number of syphilis cases during pregnancy is attributable to various factors, including changes in sexual behavior, an increase in travel and migration, limited availability of healthcare opportunities (particularly in terms of access to prenatal care), and limited awareness and education regarding maternal and obstetric services (27). A single prenatal syphilis screening may be inadequate, and more frequent testing during pregnancy is therefore necessary, even for women at a relatively low risk (28). Global authorities and guidelines from most countries recommend syphilis screening at the first prenatal visit; in some countries, additional screening of high-risk women in late pregnancy and at delivery to identify new infections is recommended.

The disease burden of syphilis in the elderly population remains a concern. GBD 2021 data demonstrated that among all older adults (aged >60), the syphilis disease burden was the highest in the 75–79-year age group. Several studies have discussed the prevalence of syphilis in older age groups. For instance, a study in Brazil explored syphilis detection rates among older adults and noted that the rate increased approximately sixfold over 2011–2019, with an average annual growth of 25% (28). The high age-standardized DALY rates observed in the elderly population may be attributable to various physiological, psychological, and emotional changes occurring in modern times. Furthermore, older adults often lack knowledge regarding sexual health, which leads them to neglect condom usage. This consequently increases syphilis transmission and infection in this population (29).

In the current study, unsafe sex was noted to be the primary risk factor for syphilis. However, the epidemiology of syphilis and the factors influencing it are complex; they include social factors, sexual education levels, medical resource allocation, and infectious disease prevention and control policy effectiveness. Syphilis infection is associated with certain behaviors and factors, such as incarceration, multiple or anonymous sexual partners, illegal drug use, and seeking sexual partners through high-risk networks (e.g., dating apps) (8). In some regions, poverty and limited medical resources may also contribute to higher syphilis infection rates. Moreover, a lack of sexual health education can render individuals without the knowledge necessary for syphilis prevention and treatment. Although syphilis can be diagnosed easily and treated using inexpensive antibiotics, it remains a major global health issue. Therefore, the development of syphilis control and prevention measures, including screening and treatment for all pregnant women and targeted interventions for high-risk populations, is warranted. Nevertheless, ongoing studies are focused on vaccine development, antibiotic prophylaxis, and digital information delivery as syphilis-preventive strategies (16).

The present study, for the first time, provides the most recent estimation of the global epidemiology of syphilis, covering 204 countries, 21 disease-burden regions, and 5 SDI regions, which had not been assessed before. However, because of a lack of comprehensive data, we could not perform an exhaustive analysis. Not all syphilis cases are reported to authorities by physicians diagnosing the disease (30). Moreover, syphilis may present with atypical manifestations that potentially remain unrecognized by less-experienced physicians, resulting in misdiagnosis and underreporting (31, 32).

## 5 Conclusion

The present results based on GBD 2021 data improve the current understanding of the global epidemiology of syphilis and provide crucial data to support the development of targeted prevention and control measures. Although various effective treatment modalities for syphilis are available clinically, early detection and control of relevant risk factors remain essential strategies for transmission prevention.

## Data Availability

The original contributions presented in the study are included in the article/Supplementary material, further inquiries can be directed to the corresponding author.
