# Supplementary material for: Global, regional, and national burden of syphilis, 1990–2021 and predictions by Bayesian age-period-cohort analysis: a systematic analysis for the global burden of disease study 2021
Source: Front Med (Lausanne). 2024 Aug 15;11:1448841. doi: 10.3389/fmed.2024.1448841 (PMC11357943; doi:10.3389/fmed.2024.1448841)
Supplement: Supplementary file 1 [file Table_1.DOCX]

Supplementary Material

**Supplementary Table 1.** Numbers and age-standardized rates (per 100,000 population) for syphilis prevalence, incidence, deaths, and DALYs in 204 countries or territories in 2021.

| Country or territory | Prevalence (95%UI) | | Incidence (95%UI) | | Deaths (95%UI) | | DALYs (95%UI) | |
| --- | --- | --- | --- | --- | --- | --- | --- | --- |
|  | Number (95% UI) | Age-standardized rate (95% UI) | Number (95% UI) | Age-standardized rate (95% UI) | Number (95% UI) | Age-standardized rate (95% UI) | Number (95% UI) | Age-standardized rate (95% UI) |
| Palestine | 14313.82 (10611.35-18357.97) | 276.94 (207.78-349.89) | 4246.46 (3113.31-5630.95) | 78.65 (58.64-102.76) | 17.05 (7.66-33.08) | 0.39 (0.19-0.72) | 1343.50 (517.25-2769.63) | 24.69 (10.19-49.89) |
| Bangladesh | 1634283.33 (1257276.23-2062566.76) | 952.35 (739.86-1184.57) | 443269.14 (329123.71-584990.23) | 251.22 (189.30-329.60) | 2041.56 (790.17-4134.98) | 1.58 (0.65-3.15) | 176610.18 (65675.76-360508.75) | 131.26 (49.11-267.02) |
| United States Virgin Islands | 609.67 (503.65-739.95) | 840.23 (673.70-1043.67) | 130.98 (103.49-163.21) | 187.58 (145.06-236.38) | 0.30 (0.14-0.56) | 0.71 (0.29-1.45) | 25.07 (11.64-48.61) | 62.96 (24.88-126.35) |
| Bosnia and Herzegovina | 5298.64 (4090.30-6650.17) | 163.88 (125.74-206.13) | 1434.27 (1073.48-1863.93) | 46.14 (34.15-60.39) | 1.26 (0.57-2.50) | 0.08 (0.03-0.17) | 100.16 (42.34-212.73) | 6.63 (2.45-14.53) |
| Tuvalu | 156.70 (113.74-212.12) | 1231.02 (899.48-1667.49) | 27.19 (20.55-35.19) | 213.89 (162.93-274.76) | 0.13 (0.04-0.29) | 0.99 (0.35-2.25) | 12.02 (4.57-26.64) | 94.24 (35.92-208.32) |
| Democratic People's Republic of Korea | 136439.71 (103243.86-174859.17) | 463.54 (351.18-592.57) | 39246.61 (29383.65-50759.05) | 136.62 (102.57-177.80) | 43.45 (16.34-103.62) | 0.29 (0.10-0.69) | 3866.19 (1421.66-8992.46) | 26.36 (9.19-61.99) |
| Timor-Leste | 7471.59 (5704.06-9395.21) | 576.37 (439.73-711.50) | 2186.87 (1598.77-2854.25) | 158.59 (120.23-204.76) | 9.55 (3.54-20.23) | 0.50 (0.19-1.07) | 860.55 (324.32-1819.91) | 44.83 (17.29-93.92) |
| Zimbabwe | 281523.97 (218514.64-358307.94) | 1916.74 (1482.25-2426.56) | 82099.94 (60862.12-106345.28) | 533.51 (404.40-683.53) | 482.95 (188.37-994.28) | 2.29 (0.94-4.65) | 42968.15 (16751.84-87536.87) | 197.79 (79.20-399.79) |
| Albania | 4362.36 (3325.86-5452.46) | 159.74 (122.05-198.97) | 1240.94 (920.03-1622.70) | 46.30 (34.12-60.62) | 1.96 (0.91-4.00) | 0.12 (0.05-0.26) | 146.30 (62.50-314.54) | 10.00 (3.79-22.26) |
| Viet Nam | 525296.72 (395645.39-684049.61) | 481.36 (365.64-620.08) | 154351.77 (117128.44-201108.62) | 145.39 (109.55-189.01) | 193.4 (68.73-401.50) | 0.26 (0.09-0.53) | 17840.12 (6678.59-36534.06) | 23.50 (8.65-48.39) |
| Taiwan (Province of China) | 108342.60 (83758.82-137839.48) | 423.95 (319.17-536.38) | 30988.61 (23701.86-40540.21) | 131.02 (98.58-171.14) | 1.81 (1.52-2.23) | 0.01 (0.00-0.01) | 141.64 (109.35-187.54) | 0.50 (0.40-0.66) |
| Republic of Korea | 191009.84 (147394.28-245865.59) | 340.30 (259.88-429.62) | 52678.14 (39285.58-68992.13) | 100.09 (74.32-129.87) | 41.72 (17.94-90.46) | 0.29 (0.10-0.64) | 3597.42 (1504.05-7716.36) | 25.57 (9.37-57.23) |
| Slovenia | 2861.49 (2193.04-3743.44) | 143.92 (107.09-185.74) | 831.12 (629.21-1083.69) | 45.13 (33.00-59.38) | 0.29 (0.20-0.45) | 0.01 (0.00-0.01) | 8.92 (6.63-12.95) | 0.27 (0.20-0.39) |
| Venezuela (Bolivarian Republic of) | 207942.23 (163485.79-260854.62) | 785.56 (613.62-981.63) | 47582.03 (36208.25-61705.34) | 184.45 (138.68-240.98) | 17.24 (12.26-24.47) | 0.07 (0.05-0.10) | 1238.28 (923.98-1704.78) | 5.10 (3.73-7.10) |
| El Salvador | 21228.70 (15753.68-27145.78) | 320.53 (240.98-407.81) | 6659.33 (4892.65-8737.11) | 98.85 (73.24-128.33) | 9.07 (3.44-20.45) | 0.16 (0.06-0.36) | 804.39 (301.70-1775.03) | 14.19 (5.25-31.39) |
| Jamaica | 26067.09 (19940.88-32861.63) | 848.82 (657.09-1058.76) | 6771.42 (5009.96-8842.77) | 218.28 (163.78-284.46) | 1.50 (1.08-2.07) | 0.06 (0.05-0.09) | 108.72 (83.74-139.31) | 5.06 (3.76-6.74) |
| Somalia | 535738.31 (430781.92-666200.71) | 2961.03 (2371.52-3624.90) | 147223.89 (112074.62-187643.13) | 761.17 (582.94-963.72） | 1473.06 (596.97-3043.49） | 3.55 (1.51-7.19） | 132376.27 (53380.29-274817.65) | 305.13 (130.18-612.02） |
| Qatar | 31413.09 (21291.75-42476.88) | 726.89 (532.34-931.51) | 7812.47 (5581.37-10655.18） | 184.60 (137.52-244.89） | 4.80 (1.78-10.36） | 0.29 (0.120.59） | 420.32 (151.15-923.53) | 22.52 (8.15-49.26） |
| Saint Lucia | 1674.76 (1326.98-2061.21） | 907.99 (715.94-1118.34） | 409.5 (310.71-526.92） | 222.63 (169.72-285.35） | 0.21 (0.16-0.28） | 0.14 (0.11-0.19） | 13.38 (10.26-17.17) | 10.49 (7.63-13.94） |
| Togo | 114042.98 (86094.14-145913.18） | 1416.82 (1082.03-1767.15） | 32880 (24563.24-42869.63） | 393.43 (295.92-509.82） | 158.73 (61.11-317.82） | 1.44 (0.58-2.81） | 14155.29 (5434.15-28227.43) | 124.38 (48.98-246.24） |
| Lesotho | 43285.63 (34078.91-54370.03） | 2240.78 (1777.37-2802.00） | 11461.79 (8596.03-14948.44） | 568.08 (432.42-728.90） | 66.73 (25.35-136.06） | 3.4 (1.32-6.86） | 5927.95 (2256.71-11988.43) | 296.95 (114.70-597.67） |
| Bolivia (Plurinational State of) | 129302.29 (99641.58-163525.52） | 1044.80 (813.66-1305.62） | 34478.68 (25660.63-45024.26） | 273.44 (205.34-354.00） | 237.90 (87.45-505.19） | 2.04 (0.75-4.31） | 21364.16 (7879.16-45361.91) | 182.21 (67.25-386.73） |
| India | 15073260.17 (11636252.03-19197463.38） | 993.52 (773.05-1254.72） | 3955480.96 (2909500.96-5191433.34） | 254.59 (189.51-331.61） | 11187.30 (4418.49-23354.50） | 1.06 (0.42-2.22） | 999922.44 (392106.92-2101906.27) | 94.19 (36.38-199.00） |
| Kuwait | 15497.79 (11391.53-20225.14） | 260.55 (196.08-329.72） | 4444.99 (3294.63-5830.76） | 77.00 (56.77-99.89） | 0.52 (0.38-0.68） | 0.02 (0.02-0.03） | 23.90 (18.60-31.40) | 0.69 (0.55-0.86） |
| Mauritania | 74790.88 (58092.33-95793.37） | 1848.29 (1426.97-2329.76） | 20000.72 (14740.85-26295.85） | 474.35 (354.26-620.67） | 138.79 (52.13-278.93） | 2.19 (0.85-4.42） | 12448.39 (4686.50-25071.34) | 194.10 (74.20-388.18） |
| Yemen | 101477.23 (75795.32-131322.18） | 323.01 (243.59-405.57） | 29391.45 (21478.14-38741.38） | 88.79 (65.94-116.94） | 129.07 (46.06-275.81） | 0.29 (0.12-0.64） | 11543.28 (4102.82-24722.12) | 25.19 (9.16-53.52） |
| Armenia | 5417.15 (4052.94-6990.69） | 168.23 (123.98-218.30） | 1595.78 (1193.85-2087.56） | 53.14 (38.60-71.04） | 0.70 (0.45-1.12） | 0.02 (0.01-0.03） | 38.52 (29.24-52.54) | 1.03 (0.79-1.39） |
| Guatemala | 65825.35 (49701.68-83626.03） | 407.06 (310.10-507.77） | 19948.29 (14728.31-26412.87） | 118.37 (89.58-154.57） | 4.62 (3.66-5.79） | 0.04 (0.03-0.04） | 360.07 (286.87-443.94) | 2.56 (2.04-3.15） |
| Belarus | 15507.06 (12210.47-19877.03） | 157 (119.30-198.77） | 4433.23 (3374.95-5672.93） | 48.64 (36.79-62.91） | 2.79 (2.10-3.63） | 0.02 (0.02-0.03） | 181.45 (143.55-226.33) | 1.76 (1.39-2.19） |
| Central African Republic | 264824.45 (212291.29-333342.31） | 5272.6 (4211.16-6584.16） | 70793.65 （53712.06-91397.10） | 1319.54 （1008.39-1701.85） | 418.58 （161.82-837.71） | 4.86 （1.97-9.53） | 37921.44 (14871.79-75585.71) | 432.8 （175.39-847.94） |
| Gambia | 38032.01 (29410.05-48241.22） | 1631.28 (1272.04-2037.78） | 9761.26 （7193.86-12868.56） | 398.41 （299.49-516.09） | 80.27 （32.09-166.15） | 2.26 （0.93-4.64） | 7202.90 (2893.61-14963.96) | 198.58 （81.33-407.57） |
| Madagascar | 877803.78 (692347.32-1105974.04） | 3291.94 (2587.96-4112.99） | 239260.33 （180937.50-314507.35） | 848.19 （651.02-1091.37） | 1760.78 （697.15-3488.00） | 4.45 （1.81-8.81） | 158851.39 (63344.32-314574.25) | 397.41 （160.37-782.03） |
| Bermuda | 316.55 (245.30-397.46） | 519.66 (388.13-655.45） | 88.83 （67.42-112.99） | 159.40 （118.33-205.32） | 0.01 （0.01-0.02） | 0.01 （0.01-0.01） | 0.84 (0.64-1.10) | 0.92 （0.71-1.19） |
| Bulgaria | 8584.22 (6462.68-11182.86） | 129.42 (96.44-166.53） | 2513.38 （1897.93-3303.04） | 41.00 （30.59-53.14） | 0.6 （0.43-0.88） | 0.00 （0.00-0.01） | 26.80 (20.75-35.39) | 0.27 （0.20-0.35） |
| Spain | 102097.06 (79337.49-130678.64） | 222.13 (165.06-285.04） | 29717.54 （22679.62-38622.20） | 69.65 （51.90-91.37） | 9.65 （7.24-12.89） | 0.01 （0.01-0.01） | 334.52 (270.21-421.27) | 0.48 （0.39-0.60） |
| Estonia | 2256.89 (1748.09-2895.36） | 174.50 (130.92-218.88） | 634.29 （482.79-842.78） | 52.26 （38.60-69.07） | 0.68 （0.50-0.93） | 0.03 （0.02-0.04） | 31.49 (24.31-40.72) | 1.72 （1.36-2.21） |
| Cuba | 54668.94 (42526.75-68487.62） | 478.30 (370.41-596.63） | 15882.43 （12295.36-19957.16） | 145.88 （112.52-185.98） | 4.08 （3.32-4.84） | 0.02 （0.02-0.03） | 212.24 (175.12-259.29) | 1.49 （1.24-1.79） |
| Fiji | 15238.22 （12079.95-18828.79） | 1612.56 （1279.58-1994.78） | 3190.51 （2399.72-4105.76） | 337.8 （254.05-434.64） | 22.39 （7.99-44.72） | 2.57 （0.92-5.13） | 2032.10 (735.60-4030.76) | 232.75 （84.20-461.71） |
| Suriname | 2500.77 （1987.48-3051.64） | 422.35 （334.31-516.01） | 716.82 （555.99-911.61） | 121.52 （93.78-155.11) | 1.08 （0.42-2.31） | 0.24 （0.09-0.53） | 97.09 (38.69-209.03) | 21.92 （8.35-47.52） |
| Saint Kitts and Nevis | 629.71 （490.86-799.40） | 981.66 （758.11-1248.89） | 146.55 (109.85-191.25） | 234.71 (174.22-306.43） | 0.04 (0.03-0.05） | 0.07 (0.05-0.09） | 2.37 (1.86-2.95) | 4.16 (3.27-5.35） |
| Nigeria | 2735141.81 （2121673.77-3469995.84） | 1261.59 （977.43-1587.91） | 725717.68 (533163.56-948815.99） | 328.65 (240.65-429.63） | 4796.92 (1704.28-9753.77） | 1.30 （0.48-2.68） | 432074.72 (155632.18-877412.24) | 113.39 (42.55-226.31） |
| Philippines | 1004047.69 （782055.58-1272089.93） | 849.73 （668.24-1064.26） | 253970.88 (185828.78-333017.35） | 209.50 (154.88-273.39） | 915.93 (334.09-1910.22） | 0.84 (0.31-1.75） | 83573.99 (31469.46-172186.03) | 76.42 (28.80-157.47） |
| Mexico | 531812.88 （407257.07-680513.79） | 387.68 （298.06-497.28） | 148610.53 (109370.78-195240.93） | 107.94 (79.62-141.16） | 43.91 (37.39-54.50） | 0.04 (0.03-0.05） | 2438.18 (2074.28-2930.16) | 2.12 (1.80-2.53） |
| Argentina | 353397.64 （274631.06-446944.25） | 749.41 （583.45-950.84） | 94277.09 (70271.40-122070.00） | 201.25 (150.35-259.61） | 37.93 (28.90-47.51） | 0.12 (0.08-0.15） | 2740.57 (1916.92-3516.93) | 9.31 （6.20-12.29） |
| Switzerland | 19782.93 （15510.67-25533.11） | 209.90 （158.16-266.59） | 5845.29 (4515.31-7582.75） | 66.86 (50.58-87.40） | 2.42 (1.78-3.20） | 0.01 (0.01-0.02） | 68.31 (54.04-87.58) | 0.48 (0.38-0.62） |
| North Macedonia | 3739.31 （2866.15-4744.08） | 163.56 （124.98-204.55） | 1043.18 (974.08-1380.77） | 46.46 (34.11-61.15） | 0.87 (0.34-1.79） | 0.08 (0.03-0.18） | 73.18 (28.54-155.65) | 7.42 (2.60-16.36） |
| Lithuania | 4647.44 （3624.55-5884.67） | 168.36 （127.07-212.26） | 1322.53 （1019.19-1707.86） | 51.37 (39.10-66.73） | 0.57 (0.43-0.81） | 0.01 (0.01-0.02） | 37.17 (28.30-48.10) | 1.00 (0.78-1.26） |
| Panama | 24170.28 (18760.28-30197.61） | 552.92 (429.56-691.77） | 6611.65 (4974.15-8596.28） | 150.48 (113.81-195.34） | 2.07 (1.60-2.65） | 0.05 (0.04-0.07） | 137.25 (109.00-173.00) | 3.64 (2.85-4.63） |
| Solomon Islands | 10542.37 (8284.89-13305.90） | 1548.01 (1220.45-1950.53） | 2254.24 (1678.57-2916.41） | 316.06 (237.14-409.74） | 29.65 (11.07-58.63） | 3.06 (1.16-6.02） | 2684.71 (1018.24-5287.00) | 276.51 (106.05-542.37） |
| Lao People's Democratic Republic | 40704.97 (30157.37-51728.65） | 523.63 (396.71-656.08） | 12017.94 (8883.51-15785.60） | 149.32 (112.21-194.88） | 29.55 (10.61-63.78） | 0.36 (0.14-0.80） | 2664.61 (970.90-5740.28) | 32.04 (11.87-68.64） |
| New Zealand | 16095.26 (12090.80-20598.40） | 304.00 (226.85-392.02） | 4562.05 (3349.51-5980.66） | 89.15 (65.06-116.91） | 0.57 (0.44-0.75） | 0.01 (0.01-0.01） | 20.59 (15.69-26.67) | 0.29 (0.22-0.41） |
| Malaysia | 156815.58 115512.82-205812.37） | 436.72 (326.28-566.28） | 49228.13 (36167.42-63753.48） | 136.5 (101.28-175.78） | 24.40 (10.43-51.89） | 0.10 (0.04-0.22） | 2200.83 (949.43-4660.24) | 9.24 (3.84-19.87） |
| Belize | 2065.71 (1550.87-2652.98） | 451.23 (342.02-573.27） | 643.55 (476.97-829.18） | 135.96 (101.82-174.17） | 0.02 (0.02-0.03） | 0.01 (0.01-0.01） | 3.69 (2.79-5.06) | 0.95 (0.72-1.27） |
| Greece | 21710.61 (16842.25-27714.21） | 218.11 (165.57-276.86） | 6302.56 (4731.95-8129.97） | 68.26 (50.14-88.67） | 1.25 （1.01-1.55） | 0.01 （0.00-0.01） | 88.81 (68.57-115.47) | 0.56 （0.43-0.72） |
| Micronesia (Federated States of) | 1467.35 （1176.35-1791.38） | 1378.09 （1113.29-1683.76） | 304.97 （231.66-396.33） | 279.62 (213.50-360.86） | 1.86 (0.73-3.78） | 2.09 （0.82-4.22） | 171.15 (69.29-343.70) | 189.80 （76.61-381.84） |
| Cabo Verde | 11542.56 （8581.47-14738.52） | 1852.75 （1390.55-2340.93） | 3284.24 （2416.00-4319.36） | 521.23 （384.39-687.30） | 4.21 (1.54-8.87） | 1.02 （0.37-2.16） | 375.13 (135.56-783.60) | 90.50 （32.33-189.78） |
| Greenland | 244.70 （186.10-319.34） | 458.07 （339.60-620.10） | 51.67 （40.61-66.66） | 92.06 （71.17-118.96） | 0.05 (0.02-0.10） | 0.12 （0.05-0.26） | 4.33 (1.70-9.04) | 11.05 （4.17-23.44） |
| Marshall Islands | 950.71 （745.26-1166.52） | 1599.24 （1258.48-1952.51） | 197.85 （149.51-253.94） | 326.12 （248.83-417.28） | 1.04 (0.39-2.15） | 1.90 (0.73-3.93） | 96.01 (37.71-195.93) | 174.55 （68.68-356.11） |
| Malta | 939.26 （717.73-1204.92） | 212.23 （155.84-266.54） | 276.38 （209.40-359.77） | 67.34 （50.38-87.86） | 0.02 (0.02-0.03） | 0.00 (0.00-0.00） | 0.85 (0.64-1.19) | 0.13 （0.09-0.21） |
| Senegal | 280460.92 （211720.09-362230.93） | 1954.97 （1476.99-2501.58） | 82676.61 （60427.92-108395.82） | 531.94 (391.88-689.29） | 205.10 (78.78-413.66） | 0.98 (0.39-1.91） | 18036.41 (6981.35-36506.77) | 81.22 （32.21-161.87） |
| Botswana | 62825.96 （48754.90-80715.52） | 2385.42 （1867.28-3039.53） | 17416.03 （12850.92-22547.62） | 648.35 （484.46-834.05） | 53.41 (20.13-110.78） | 2.32 (0.88-4.79） | 4794.04 (1834.29-9934.74) | 206.14 （78.88-426.99） |
| Tokelau | 25.63 （18.66-35.41） | 1891.54 （1377.82-2607.74） | 3.39 （2.64-4.27） | 275.52 （213.93-347.24） | 0.01 (0.01-0.03） | 1.72 (0.63-3.72） | 1.41 (0.58-2.95) | 160.42 （62.66-339.87） |
| American Samoa | 567.48 （458.79-692.76） | 1158.3 （926.40-1427.92） | 112.99 （87.60-143.58） | 241.5 （186.82-308.89） | 0.62 （0.24-1.26） | 1.90 （0.73-3.87） | 56.88 (22.54-114.26) | 173.31 （67.67-349.72） |
| Republic of Moldova | 8483.26 （6571.33-10772.14） | 218.89 （167.16-274.01） | 2225.43 （1702.65-2835.11） | 60.85 （45.57-78.03） | 1.18 （0.93-1.53） | 0.03 （0.02-0.04） | 74.91 (59.75-92.43) | 1.95 （1.58-2.45） |
| Angola | 1241905.68 （962144.00-1589801.74） | 4448.27 （3453.22-5584.24） | 363676.77 （269149.01-475422.48） | 1225.80 （918.41-1589.26） | 1841.66 （686.41-3668.75） | 3.31 （1.29-6.54） | 166096.99 (62294.76-329377.65) | 293.8 （114.29-578.19） |
| Maldives | 4197.69 （3095.88-5567.32） | 615.35 （476.33-782.70） | 1216.01 （885.99-1624.85） | 176.41 （131.24-229.96） | 0.82 （0.34-1.75） | 0.28 （0.11-0.59） | 72.87 (30.31-151.71) | 24.03 （9.38-50.98） |
| Poland | 59412.5 （44970.45-77220.66） | 152.78 （112.66-198.04） | 16376.72 （12126.50-21721.71） | 45.23 （32.87-59.74） | 1.32 （1.08-1.78） | 0.00 （0.00-0.00） | 128.80 (97.93-167.56) | 0.24 （0.18-0.31） |
| Vanuatu | 4511.36 （3621.23-5637.90） | 1434.92 （1144.39-1788.34） | 948.15 （716.81-1243.27） | 292.18 （221.82-383.47） | 10.78 （4.08-22.37） | 2.59 （0.99-5.34） | 979.85 (375.59-2022.90) | 234.75 （90.63-482.69） |
| Romania | 34627.31 （26629.84-43953.17） | 190.23 （143.67-239.17） | 9159.39 （6956.48-11882.19） | 53.65 （40.24-69.81） | 1.75 （1.25-2.65） | 0.01 （0.00-0.01） | 83.00 (62.87-110.68) | 0.33 （0.25-0.44） |
| Sierra Leone | 117558.20 （87781.26-150533.91） | 1398.41 （1067.18-1749.75） | 34870.49 （25866.68-45777.86） | 390.93 （296.30-505.66） | 168.15 （58.45-358.09） | 1.25 （0.46-2.60） | 15025.18 (5256.98-31554.52) | 107.85 （39.24-226.77） |
| United Arab Emirates | 33841.29 （24324.72-46182.00） | 244.02 （187.13-311.36） | 9490.49 （6840.01-13145.58） | 73.13 （55.33-95.09） | 2.20 （0.96-4.72） | 0.07 （0.03-0.14） | 197.59 (86.29-417.99) | 5.10 （2.15-10.99） |
| Ireland | 10702.35 （8259.57-13648.43） | 214.63 （161.73-271.34） | 3213.20 （2383.64-4111.85） | 67.41 （50.29-87.30） | 0.32 （0.25-0.41） | 0.00 （0.00-0.01） | 15.23 (11.63-19.68) | 0.23 （0.17-0.32） |
| Namibia | 53779.00 （41428.77-69301.48） | 2141.63 （1660.70-2699.02） | 15102.35 （11316.11-19832.68） | 581.32 （443.55-753.19） | 71.22 （27.27-150.81） | 2.63 （1.03-5.52） | 6363.13 (2422.21-13492.42) | 232.76 （89.80-491.29） |
| Samoa | 1010.01 （802.98-1231.14） | 491.32 （389.80-604.73） | 271.37 （207.18-350.12） | 132.24 （100.85-171.64） | 0.82 （0.30-1.74） | 0.29 （0.12-0.62） | 74.64 (27.95-157.51) | 25.83 （10.14-53.55） |
| Turkmenistan | 11130.73 （8265.04-14245.19） | 206.33 （154.59-261.95） | 3379.94 （2518.71-4444.25） | 62.20 （46.47-81.53） | 0.92 （0.58-1.39） | 0.02 （0.01-0.03） | 66.73 (48.54-93.49) | 1.36 （1.00-1.90） |
| Cook Islands | 245.58 （189.65-317.53） | 1563.12 （1178.21-2048.81) | 41.05 （31.13-51.93） | 261.68 （198.86-334.62） | 0.09 （0.04-0.17） | 0.84 （0.35-1.64） | 8.25 (3.45-15.81) | 76.33 （31.31-147.54) |
| Congo | 220936.50 （168149.09-275942.67） | 4122.26 （3153.74-5094.42） | 65856.73 （49450.79-84825.79） | 1178.56 （890.24-1516.13） | 162.62 （62.29-322.46） | 2.72 （1.07-5.36） | 14668.51 (5701.80-29131.23) | 241.71 （94.70-477.64） |
| Norway | 15289.16 （11960.16-19103.33） | 264.49 （201.03-332.11） | 4019.80  （3077.50-5209.15） | 75.38 （56.63-98.90） | 2.10 （1.70-2.60） | 0.02 （0.01-0.02） | 113.75 (85.19-148.82) | 1.35 （0.98-1.82） |
| Guam | 1447.30 （1145.25-1793.88） | 944.90 （740.36-1175.68） | 347.83 （266.65-451.12） | 227.69 （174.08-295.97） | 1.92 （0.69-3.99） | 1.50 （0.54-3.12） | 173.75 (63.90-358.35) | 135.84 （49.59-280.83） |
| Russian Federation | 296957.62 （233072.88-375447.42） | 195.61 （150.41-248.28） | 77797.32 （60100.52-101074.63） | 55.25 （41.45-72.23） | 41.10 （35.79-50.61） | 0.02 （0.02-0.03） | 2613.96 (2167.82-3166.07) | 1.53 （1.29-1.81） |
| Belgium | 24704.73 （19017.40-31655.64） | 215.97 （161.62-274.71） | 7310.18 （5521.08-9566.74） | 67.89 （50.82-89.81） | 1.49 （1.19-1.89) | 0.01 （0.01-0.01) | 55.27 (44.38-69.14) | 0.32 （0.26-0.42) |
| Austria | 20196.23 （15566.77-25921.90） | 217.02 （163.43-278.07） | 6002.69 （4556.01-7722.80） | 68.47 （50.79-89.91） | 0.95 （0.77-1.18) | 0.01 （0.00-0.01) | 49.22 (39.16-61.97) | 0.37 （0.29-0.47) |
| Ukraine | 80231.84 （61875.37-102040.68） | 175.90 （133.39-225.18） | 21085.59 （15894.72-27705.17） | 50.27 （37.18-66.54） | 8.73 （6.02-12.68) | 0.01 （0.01-0.02) | 617.56 (454.48-803.16) | 0.97 （0.71-1.25) |
| Saint Vincent and the Grenadines | 772.79 （617.49-946.84） | 668.47 （531.63-830.65） | 200.12 （153.09-257.58) | 174.67 （132.98-226.91） | 0.24 （0.18-0.31) | 0.21 （0.16-0.27) | 12.35 (9.79-15.36) | 12.36 （9.73-15.72) |
| Papua New Guinea | 124142.96 （97773.08-157999.64） | 1158.72 （907.43-1459.43） | 27133.52 （20313.70-35736.63 | 243.43 （183.36-319.89） | 469.77 （171.73-930.57) | 2.92 （1.08-5.76) | 42471.37 (15711.88-83880.01) | 262.73 （98.37-516.35) |
| Mongolia | 23184.31 （18020.20-29667.95） | 673.27 （521.38-867.98） | 3694.31 （2788.39-4712.96) | 111.09 （82.31-142.87） | 42.26 （14.16-93.64) | 1.16 （0.40-2.56) | 3742.73 (1248.63-8349.65) | 102.05 （34.31-226.91) |
| Montenegro | 1165.12 （932.00-1448.50） | 199.68 （156.53-251.70） | 286.98 （221.44-373.33) | 48.56 （36.88-63.12) | 0.34 （0.16-0.66) | 0.08 （0.03-0.17) | 26.96 (11.37-54.45) | 7.29 （2.76-15.26) |
| Peru | 288867.87 （219437.24-364120.11） | 748.12 （572.36-938.34） | 84987.19 （63701.83-109407.21) | 218.33 （164.35-279.60) | 235.45 （86.90-529.47) | 0.72 （0.27-1.63) | 20889.72 (7619.11-47262.07) | 64.22 （23.33-145.45) |
| Brazil | 1816379.96 （1513760.23-2202313.16) | 795.58 （666.24-961.80) | 435006.09 （330344.02-546364.64) | 191.00 （145.63-239.23) | 275.24 （206.49-328.55) | 0.15 （0.11-0.18) | 20639.38 (14653.18-25217.19) | 11.89 （8.15-14.65) |
| Uganda | 1178000.29 （945252.44-1471942.21) | 2967.93 （2371.58-3649.42) | 301601.43 （225459.74-393243.54) | 728.63 （554.13-929.97) | 2934.72 （1130.02-5705.16) | 3.99 （1.56-7.67) | 265095.46 (103240.26-513823.21) | 357.70 （142.64-685.87) |
| Tonga | 843.62 （673.43-1047.86） | 819.30 （645.96-1013.24） | 198.92 （151.77-255.82） | 194.8 （147.57-252.78） | 1.26 （0.44-2.59） | 0.89 （0.32-1.83） | 114.04 (41.01-232.87) | 80.35 （29.45-163.25） |
| Japan | 397043.25 （309251.03-506827.09） | 333.94 （256.35-422.56） | 106313.57 （79596.89-138596.36） | 97.4 （71.84-127.66） | 34.75 （26.97-41.46） | 0.01 （0.01-0.01） | 957.48 （779.10-1165.73) | 0.39 （0.29-0.52） |
| Honduras | 36741.59 （27679.00-46566.87） | 362.13 （280.28-451.66） | 11357.01 （8377.17-14833.87） | 105.87 （80.15-136.40) | 24.06 （10.39-50.23) | 0.24 （0.11-0.49) | 2034.27 （820.84-4350.87) | 19.57 （8.16-41.57) |
| Colombia | 280654.01 （207451.10-360824.77） | 526.44 （392.03-674.73） | 80536.21 （59214.59-106891.23) | 151.20 （111.40-199.46) | 35.41 （26.75-46.98) | 0.08 （0.06-0.11) | 2198.06 （1632.60-2988.12) | 5.66 （3.99-7.96) |
| Cyprus | 3579.05 （2721.52-4571.49) | 246.59 （188.88-304.63) | 1006.23 （751.89-1327.97) | 70.59 （53.29-92.27) | 0.75 （0.37-1.43) | 0.08 （0.03-0.17) | 53.82 （22.69-108.96) | 6.73 （2.52-14.09) |
| Guyana | 3333.94 （2551.48-4191.09) | 416.25 （320.09-519.67) | 1016.28 （766.00-1316.40) | 124.17 （94.95-159.11) | 0.55 （0.40-0.74) | 0.08 （0.06-0.10) | 38.03 （28.78-48.76) | 5.15 （3.89-6.59) |
| Haiti | 163979.10 （130437.95-204517.48) | 1219.29 （981.30-1506.01) | 36037.66 （27095.29-46158.48) | 259.16 （196.35-332.82) | 521.28 （190.37-1081.92) | 3.23 （1.21-6.71) | 47025.90 （17284.73-97480.25) | 290.44 （108.41-600.14) |
| Portugal | 22938.80 （17572.69-29436.04) | 220.02 （165.51-280.24) | 6628.69 （5032.92-8621.86) | 68.21 （51.50-90.12) | 1.72 （1.33-2.26) | 0.01 （0.01-0.01) | 70.21 （54.62-88.66) | 0.41 （0.32-0.52) |
| Canada | 87146.36 （66610.43-111202.98) | 226.6 （168.47-292.44) | 25828.65 （19698.17-34006.35) | 72.03 （53.71-94.89) | 5.03 （4.07-6.11 | 0.01 （0.01-0.01) | 263.12 （205.59-335.51) | 0.50 （0.40-0.63) |
| Syrian Arab Republic | 37663.01 （29632.20-46437.73) | 278.04 （209.83-352.72) | 10792.61 （8109.09-13920.64) | 77.57 （57.43-100.53) | 32.19 （13.37-68.49) | 0.34 （0.14-0.72) | 2729.10 （1055.80-5889.83) | 28.51 （10.89-61.64) |
| Bhutan | 12591.79 （9498.40-15951.33) | 1479.02 （1143.12-1831.59) | 3106.70 （2295.20-4057.57) | 354.08 （265.20-456.59) | 17.57 （6.16-38.34) | 2.93 （1.03-6.39) | 1577.17 （563.23-3447.29） | 262.24 （93.09-574.16) |
| Egypt | 224686.93 （169135.76-286454.17) | 213.13 （161.20-269.65) | 66567.78 （48769.84-87456.96) | 62.05 （45.73-81.18) | 268.25 （101.01-598.04) | 0.24 （0.10-0.50) | 23337.36 （8352.75-53116.23) | 19.12 （7.10-43.00) |
| Mali | 479863.93 （378810.29-600153.05) | 2246.35 （1753.19-2805.30) | 117093.95 （86472.93-152995.51) | 520.91 （388.90-680.34) | 1744.33 （650.00-3612.36) | 3.60 （1.36-7.33) | 156987.00 （59138.87-325266.02) | 318.21 （122.66-650.92) |
| Sudan | 303066.53 （230608.44-389089.35) | 701.27 （541.51-882.47) | 64465.22 （46964.14-86544.68) | 139.16 （103.38-183.97) | 696.33 （241.91-1487.22) | 1.27 （0.45-2.71) | 62766.25 （21954.84-133718.38) | 114.13 （40.28-242.80) |
| Seychelles | 743.85 （587.32-908.55) | 683.43 （542.20-844.55) | 185.54 （142.85-235.19) | 168.94 （129.09-213.01) | 0.18 （0.09-0.35) | 0.22 （0.10-0.44） | 15.17 （6.83-29.45) | 18.79 （7.89-37.10） |
| Netherlands | 36898.99 （28296.37-46769.90) | 215.58 （160.22-274.01) | 11046.66 （8320.80-14308.38) | 68.21 （50.43-88.77) | 2.51 （1.96-3.23) | 0.01 （0.01-0.01) | 83.23 （66.58-102.09) | 0.32 （0.25-0.40） |
| Uruguay | 23083.42 （17628.59-28889.66) | 682.36 （516.05-864.07) | 6294.91 （4680.10-8243.40) | 190.58 （140.56-248.60) | 2.67 （2.18-3.16) | 0.08 （0.06-0.10) | 132.12 （107.53-157.31) | 5.28 （4.15-6.56） |
| Costa Rica | 21345.99 （15977.94-26950.45) | 414.21 （310.27-522.90) | 6251.10 （4712.34-8122.14) | 122.87 （92.01-159.72) | 3.38 （2.77-4.18) | 0.08 （0.06-0.09) | 178.25 （149.35-212.61) | 4.70 （3.87-5.64) |
| Djibouti | 29423.52 （22592.93-37046.41) | 2209.58 （1728.40-2724.27) | 8228.54 （6108.32-10764.75) | 602.56 （451.00-786.38) | 49.5 （19.93-795.51) | 3.50 （1.46-6.73) | 4437.20 （1783.93-8605.45) | 308.81 （125.95-595.72） |
| Brunei Darussalam | 1878.88 （1467.74-2342.71) | 365.61 （293.36-446.91) | 517.55 （382.30-665.59) | 96.58 （72.29-123.13) | 0.65 （0.27-1.27） | 0.22 （0.09-0.43) | 54.94 （21.21-110.70) | 18.04 （6.71-36.81) |
| Thailand | 482234.43 （370606.33-616496.21) | 674.33 （507.48-864.47) | 130577.67 （98327.61-171446.87) | 197.23 （145.63-258.59) | 70.79 （29.78-148.02) | 0.25 （0.09-0.55) | 6356.71 （2762.66-13340.83) | 22.58 （8.65-49.23) |
| Oman | 13889.04 （9919.92-18749.97) | 231.03 （173.07-296.56) | 4187.40 （3051.36-5575.59) | 70.56 （53.04-91.54) | 2.58 （1.02-5.58) | 0.08 （0.04-0.16) | 212.68 （80.27-469.01) | 5.71 （2.28-12.40) |
| Cameroon | 800165.18 （615612.58-1027097.94） | 2695.24 （2082.87-3404.00） | 203847.96 （150801.34-267299.03） | 646.65 （486.42-852.01） | 1500.37 （567.68-2947.56） | 3.10 （1.22-6.07) | 135056.80 （51308.27-265261.24) | 276.18 （107.27-538.66) |
| Finland | 11787.73 （9122.38-15142.86) | 219.20 （163.62-280.03) | 3446.18 （2579.93-4487.00) | 68.84 （50.46-90.37) | 2.13 （1.70-2.64) | 0.02 （0.01-0.02) | 48.13 （38.76-59.02) | 0.50 （0.40-0.62) |
| Pakistan | 2236582.29 (1712391.75-2881161.77) | 926.89 (717.77-1173.64) | 570962.87 (409759.83-753706.37) | 230.63 (169.32-303.43) | 4246.1 (1499.75-8727.84) | 1.47 (0.53-3.00) | 383474.60 （139308.00-783249.70) | 130.58 (48.08-265.21) |
| South Sudan | 340194.38 (273805.85-420734.39） | 4095.13 (3270.86-5059.67） | 88444.79 (66741.07-114919.22） | 995.57 （754.26-1275.15） | 1240.21 （458.32-2567.19） | 7.08 （2.76-14.61） | 111512.19 （40474.31-230259.87) | 628.48 (240.55-1292.40) |
| Guinea | 227535.06 (178280.30-288761.12） | 1870.38 (1469.79-2326.26） | 58133.54 (43250.44-75069.21） | 456.99 （345.19-589.61） | 736.93 （284.59-1485.18） | 3.22 （1.30-6.37） | 66228.43 （25681.91-133674.01) | 284.79 (112.85-568.07) |
| United States of America | 1312511.98 (1025766.41-1647996.82) | 390.37 (298.13-494.63） | 352848.89 (266469.96-457847.35） | 110.18 （81.92-143.53） | 58.68 （50.95-67.71） | 0.01 （0.01-0.01） | 3537.30 （2850.33-4439.04) | 0.81 (0.66-1.00) |
| Niger | 199060.12 (148649.23-255556.21） | 1065.29 (801.68-1342.01） | 65355.81 (47786.32-84260.03） | 318.38 （239.21-409.59） | 238.79 （94.45-497.60） | 0.56 （0.22-1.16） | 20943.41 （8281.79-42565.77) | 41.72 （17.53-83.61) |
| Comoros | 18399.84 (14366.84-23020.34） | 2432.68 (1907.69-3009.70） | 5328.93 (4010.56-6942.53） | 683.28 （517.87-879.12） | 21.9 （8.52-43.79） | 2.74 （1.07-5.45） | 1960.05 （775.65-3922.72) | 242.35 （96.48-483.18) |
| Burkina Faso | 257710.93 (196107.90-326095.77) | 1299.96 (999.05-1634.53） | 75228.16 （55738.15-97279.04） | 361.13 （270.88-466.54） | 672 （258.65-1325.97） | 1.60 （0.63-3.27） | 60043.00 （23277.11-118927.52) | 137.43 （54.33-270.16) |
| Slovakia | 7755.90 (5961.44-10067.93) | 139.62 (105.27-175.79） | 2236.35 （1687.01-2886.16） | 42.52 （31.77-55.09） | 1.58 （0.79-2.98） | 0.05 （0.02-0.09） | 123.19 （57.45-240.75) | 3.96 （1.60-8.22） |
| Nepal | 314261.71 (237706.35-404924.11) | 964.26 (738.02-1221.27） | 86998.65 （63269.27-113569.66） | 256.85 （190.71-332.33） | 461.81 （172.14-967.47） | 1.51 （0.57-3.15） | 41381.73 （15451.29-86811.82) | 134.50 （50.33-281.95） |
| Liberia | 163361.32 (130435.92-203920.06) | 2986.05 (2368.81-3701.83） | 35689.75 （26441.34-45752.45） | 622.87 （466.28-797.54） | 360.35 （134.72-743.52） | 4.70 （1.82-9.61） | 32565.80 （12232.62-66988.91) | 422.51 （160.30-864.57） |
| Rwanda | 282571.81 (218583.60-358717.12) | 2217.80 (1739.99-2724.09) | 85892.71 (64333.18-110873.29) | 641.35 (487.11-815.70) | 294.72 (114.69-573.49 | 1.74 (0.71-3.37) | 26385.87 （10399.41-51418.08) | 152.18 (61.230293.45) |
| Gabon | 74357.34 (56531.64-93618.52) | 4163.12 (3187.11-5107.36) | 22139.52 (16584.40-29133.85) | 1186.16 (892.60-1536.27) | 57.27 (21.61-120.22) | 2.81 (1.07-5.85) | 5150.04 （1954.79-10814.39) | 250.63 (95.93-524.35) |
| United Republic of Tanzania | 1372353.71 (1078685.23-1720645.35) | 2541.67 (2003.45-3159.99) | 382621.89 (289864.11-497093.89) | 674.18 (517.08-861.23) | 4007.7 (1571.58-8237.18) | 4.52 (1.79-9.19) | 360405.56 （141983.92-739755.67) | 403.98 (160.61-824.93) |
| Kiribati | 1863.31 (1504.57-2300.05) | 1490.57 (1211.71-1832.34) | 370.39 (283.21-481.28) | 289.52 (223.02-377.76) | 3.26 (1.19-6.57) | 2.34 (0.87-4.68) | 299.08 （114.57-596.45) | 214.01 (82.81-425.28) |
| Nauru | 260.66 (200.57-329.84) | 2171.01 (1691.35-2731.73) | 40.15 (30.36-51.82) | 333.05 (258.13-426.46) | 0.33 (0.13-0.68) | 2.39 (0.93-4.88) | 30.85 （12.54-62.13) | 223.93 (92.33-448.00) |
| Singapore | 20145.2 (15132.02-26215.21) | 303.45 (226.85-384.07) | 5704.25 (4245.61-7527.86) | 91.64 (68.67-119.82) | 1.06 (0.81-1.36) | 0.01 (0.01-0.02) | 46.15 （36.36-58.47) | 0.58 (0.46-0.73) |
| Sweden | 24498.83 (18548.26-31694.45) | 241.36 (179.59-310.80) | 6939.63 (5156.44-9070.75) | 72.49 (53.34-94.52) | 0.80 (0.64-0.98) | 0.00 (0.00-0.00) | 35.10 （27.10-46.00) | 0.22 (0.16-0.33) |
| Germany | 183732.59 (142717.87-234199.46) | 215.15 (160.99-272.74) | 54118.08 (41157.90-70041.14) | 68.30 (50.85-89.25) | 12.59 (9.90-16.45) | 0.01 (0.01-0.01) | 409.14 （331.29-506.65) | 0.3 (0.24-0.39)0 |
| Georgia | 8969.26 (6945.99-11275.21) | 255.59 (193.92-323.42) | 2332.95 (1761.03-2991.83) | 71.14 (52.43-92.43) | 0.68 (0.52-0.95) | 0.02 (0.01-0.02) | 54.19 （42.43-67.60) | 1.63 (1.24-2.04) |
| United Kingdom | 195998.21 (153269.34-242630.05) | 299.44 (238.06-367.48) | 50183.97 (38172.54-64702.22) | 77.99 (58.50-100.98) | 59.86 (27.46-115.17) | 0.15 (0.06-0.32) | 4730.25 （1820.96-9712.16) | 13.26 (4.65-28.14) |
| Denmark | 12323.67 (9431.92-15862.75) | 205.15 (152.93-263.26) | 3646.16 (2726.01-4812.98) | 64.91 (48.09-86.19) | 2.26 (1.84-2.80) | 0.02 (0.01-0.02) | 49.97 （41.37-61.17) | 0.50 (0.41-0.62) |
| Trinidad and Tobago | 6635.70 (5012.93-8373.10) | 456.45 (341.10-574.20) | 1922.47 (1456.33-2504.97) | 138.27 (103.48-179.81) | 0.25 (0.18-0.34) | 0.02 (0.01-0.02) | 20.97 （16.27-26.14) | 1.43 (1.12-1.80) |
| Zambia | 619418.94 (485253.58-779325.81) | 3451.95 (2712.90-4239.07) | 170730.51 (129082.71-219776.15) | 892.31 (679.35-1136.52) | 1113.79 (436.93-2352.64) | 3.94 (1.57-8.25) | 100279.03 （39655.35-211709.94) | 351.10 (140.61-735.95) |
| Sao Tome and Principe | 3023.60 (2317.50-3856.23) | 1397.26 (1072.19-1760.97) | 876.34 (648.11-1143.15) | 392.38 (294.19-509.20) | 2.34 (0.88-5.05) | 1.02 (0.39-2.23) | 210.18 （80.61-431.51) | 89.40 (34.66-186.57) |
| Eritrea | 139849.18 (109248.72-177388.47) | 2205.38 (1742.57-2745.06) | 42959.94 (32189.84-54981.06) | 642.19 (485.21-819.16) | 171.76 (67.07-362.83) | 1.95 (0.80-3.97) | 15332.15 （5901.46-32488.70) | 168.43 (67.87-352.12) |
| Turkey | 204657.83 (153485.10-256487.14) | 234 (177.13-295.06) | 52627.32 (38692.85-68805.70) | 60.76 (44.55-79.59) | 186.54 (68.68-407.52) | 0.38 (0.14-0.83) | 16558.10 （5972.86-36482.62) | 33.62 (12.00-74.31) |
| Bahrain | 7009.90 (5281.40-8914.37) | 382.72 (294.11-477.91) | 1880.61 (1413.61-2455.67) | 100.14 (75.40-129.45) | 2.97 (1.09-6.33) | 0.35 (0.13-0.75) | 263.24 （92.77-567.74) | 30.61 (10.70-66.17) |
| Nicaragua | 25721.49 (19126.92-33033.70) | 365.41 (275.17 461.72) | 7955.04 (5841.02 10378.79) | 110.69 (82.45-143.62) | 14.71 (5.27-32.60) | 0.24 (0.09-0.53) | 1323.70 （478.43-2932.17) | 21.42 (7.75-47.41) |
| Bahamas | 2968.41 (2278.98-3721.18) | 706.36 (542.99-888.20) | 810.70 (608.04-1037.97) | 192.91 (143.81-248.91) | 0.09 (0.07-0.11) | 0.02 (0.02-0.03) | 7.29 （5.78-9.20) | 1.93 (1.55-2.42) |
| Italy | 140624.87 (107883.65-177325.73) | 242.58 (184.09-311.21) | 38418.69 (28621.75-50219.54) | 72.15 (52.40-95.13) | 5.58 (4.42-7.21) | 0.00 (0.00-0.00) | 248.69 （193.46-315.81) | 0.26 (0.20-0.36) |
| Barbados | 1255.63 (978.01-1556.56) | 419.40 (322.46-516.27) | 358.58 (276.33-458.05) | 123.79 (93.55-158.93) | 0.10 (0.07-0.13) | 0.03 (0.02-0.04) | 6.14 （4.71-7.86) | 2.25 (1.73-3.04) |
| Saudi Arabia | 111185.77 (80068.96-145701.28) | 225.14 (167.31-287.47) | 33698.94 (24590.68-45093.69) | 69.07 (51.35-90.40) | 19.72 (7.03-45.68) | 0.09 (0.03-0.20) | 1804.62 （652.16-4129.17) | 7.95 (2.84-18.32) |
| Iraq | 118932.42 (89250.30-155428.80) | 278.01 (211.34-360.07) | 34615.85 (25048.25-45601.44) | 78.30 (57.25-102.73) | 156.41 (56.72-333.90) | 0.40 (0.15-0.84) | 13950.80 （4987.26-29932.77) | 34.94 (12.56-74.80) |
| Northern Mariana Islands | 642.77 (509.68-813.40) | 1390.71 (1084.52-1778.18) | 118.86 (92.40-151.02) | 259.20 (201.34-329.99) | 0.27 (0.10-0.57) | 0.94 (0.35-1.98) | 25.42 （10.20-52.28) | 86.12 (33.62-179.10) |
| Palau | 171.51 (132.19-222.06) | 1046.28 (771.96-1400.69) | 32.91 (25.50-42.63) | 193.02 (149.29-247.43) | 0.06 (0.02-0.13) | 0.71 (0.26-1.49) | 6.14 （2.65-12.47) | 66.29 (26.10-138.05) |
| Andorra | 312.85 (239.26-406.35） | 454.92 (330.15-634.54） | 66.86 (50.85-84.46） | 86.35 (65.00-112.74） | 0.03 (0.01-0.06） | 0.10 (0.03-0.24） | 2.42 （1.00-5.19) | 9.22 (3.02-21.44） |
| France | 149674.54 (115586.08-187077.07） | 235.27 (176.49-299.61) | 43266.03 (32174.98-56095.73) | 72.18 (53.20-95.42) | 8.33 (6.72-10.59) | 0.01 (0.00-0.01) | 293.99 （236.33-369.21) | 0.30 (0.23-0.39) |
| Antigua and Barbuda | 535.61 (412.90-660.82） | 555.95 (427.97-687.95） | 150.75 (114.82-193.85) | 157.28 (118.79-201.13) | 0.05 (0.04-0.06) | 0.05 (0.04-0.07) | 2.75 （2.33-3.20) | 3.19 (2.71-3.70） |
| Kyrgyzstan | 12204.31 (9092.04-15823.81） | 175.05 (131.23-226.82） | 3859.40 (2812.84-5091.49) | 54.93 (40.30-72.06) | 2.33 (1.68-2.91) | 0.03 (0.03-0.04) | 197.03 （145.34-245.17) | 2.81 (2.12-3.45) |
| Tunisia | 33653.04 (25428.19-42640.15） | 272.08 (205.37-341.02） | 9359.37 (7032.28-12200.13） | 77.81 (57.86-100.97） | 15.44 (5.78-32.43） | 0.19 (0.07-0.40） | 1354.15 （490.07-2883.87) | 16.61 (5.94-35.51） |
| Mauritius | 7656.81 (5841.34-9723.99） | 564.39 (427.65-718.34） | 2127.88 (1594.79-2791.83） | 162.30 (120.99-212.87） | 0.53 (0.43-0.67） | 0.04 (0.03-0.04） | 26.41 (22.06-31.21) | 2.09 (1.78-2.45） |
| South Africa | 1707774.50 (1346243.78-2170514.74） | 2770.43 (2196.01-3478.63） | 405123.02 (299428.34-534038.12） | 652.25 (486.96-850.32） | 1517.17 (599.29-3139.26） | 3.19 (1.26-6.62） | 136633.76 (54282.19-282343.59) | 286.66 (113.25-594.36） |
| Indonesia | 3329490.76 (2599430.91-4158096.42） | 1115.87 (883.53-1388.07） | 771419.92 (576211.50-1014314.39） | 253.69 (189.76-331.61） | 3083.26 (1101.98-6303.47） | 1.45 (0.52-2.97） | 281459.02 (103609.95-571824.20) | 131.50 (47.68-268.23） |
| Chile | 99323.18 (74716.15-126846.68） | 493.84 (372.01-626.60） | 29419.50 (21716.60-38729.55） | 149.99 (110.62-196.74） | 5.49 (4.37-6.70） | 0.03 (0.02-0.04） | 333.43 (265.05-397.57) | 2.34 (1.70-2.81） |
| Israel | 19800.63 (15012.54-24967.96） | 215.87 (161.91-274.04） | 6114.47 (4600.54-7901.18） | 67.41 (50.60-87.21） | 0.54 (0.42-0.71） | 0.00 (0.00-0.01） | 26.57 (20.60-34.96) | 0.25 (0.19-0.34） |
| Czechia | 15065.93 (11576.45-19372.48） | 145.69 (108.55-185.47） | 4335.53 (3244.76-5648.59） | 45.23 (33.35-58.95） | 1.41 (0.98-2.05） | 0.01 (0.00-0.01） | 66.42 (49.93-90.29) | 0.41 (0.31-0.56） |
| Luxembourg | 1601.52 (1220.89-2038.48） | 223.56 (169.05-283.58） | 473.68 (362.78-612.75） | 70.11 (53.22-91.45） | 0.11 (0.08-0.14） | 0.01 (0.01-0.01） | 4.03 (3.19-4.98) | 0.44 (0.35-0.55） |
| Dominica | 755.72 (609.44-925.38） | 1139.75 (916.76-1415.89） | 158.61 (122.01-204.01） | 236.80 (181.74-304.47） | 0.48 (0.19-1.02） | 1.41 (0.50-2.98） | 42.05 (16.79-86.20) | 125.95 (45.45-262.51） |
| Latvia | 3282.26 (2524.37-4127.96） | 180.00 (134.84-223.18） | 894.11 (675.67-1164.83） | 52.62 (39.29-68.42） | 1.33 (0.98-1.89） | 0.05 (0.03-0.06） | 63.50 (50.43-80.93) | 2.69 (2.18-3.39） |
| Australia | 76012.33 (57864.92-97467.58） | 287.94 (217.66-363.33） | 21622.57 (16377.38-28013.38） | 86.09 (64.40-112.41） | 5.27 (4.19-6.68） | 0.01 (0.01-0.01） | 150.86 (123.46-184.86) | 0.45 (0.36-0.56） |
| China | 7919404.89 (5980309.97-10217268.34） | 533.53 (402.99-685.78） | 2047248.67 (1540693.56-2677248.79） | 146.44 (106.79-193.50） | 1991.49 (810.36-4262.32） | 0.35 (0.13-0.77） | 176653.09 (69620.35-381754.77) | 31.47 (11.71-69.21） |
| Grenada | 1004.47 (786.58-1263.42） | 920.18 (720.40-1153.20） | 251.49 (186.77-327.79） | 228.04 (169.66-294.56） | 0.10 (0.08-0.12） | 0.09 (0.08-0.12） | 5.80 (4.74-6.92) | 5.94 (4.91-7.10） |
| Iceland | 788.55 (589.13-1009.32） | 218.05 (160.56-276.33） | 240.97 (179.57-314.62） | 69.49 (51.26-90.22） | 0.03 (0.03-0.04） | 0.01 (0.00-0.01） | 1.32 (1.06-1.67) | 0.28 (0.22-0.37） |
| Puerto Rico | 17146.79 (13190.42-21418.00） | 543.55 (412.58-687.37） | 4836.00 (3658.78-6239.56） | 160.81 (121.24-208.79） | 1.51 (1.15-2.01） | 0.04 (0.03-0.05） | 78.94 (64.21-96.96) | 2.64 (2.13-3.21） |
| Iran (Islamic Republic of) | 192629.65 (145429.03-250919.60） | 208.05 (160.98-264.32） | 45917.42 (34119.57-60867.81） | 49.99 (37.30-66.28） | 111.81 (42.06-237.06） | 0.22 (0.08-0.47） | 9899.94 (3559.00-21300.52) | 19.47 (6.77-42.17） |
| Tajikistan | 24435.04 (18550.37-30960.28） | 244.30 (189.45-302.99） | 7256.36 (5513.10-9356.90） | 69.73 (53.40-89.00） | 17.86 (8.01-35.38） | 0.16 (0.08-0.30） | 1534.49 (698.00-3087.12) | 12.54 (6.26-23.92） |
| Dominican Republic | 96785.80 (76807.44-121829.66） | 832.86 (666.81-1036.30） | 25847.96 (19559.89-33628.17） | 218.92 (167.18-282.70） | 132.67 (61.05-261.42） | 1.29 (0.59-2.54） | 11041.58 4728.25-22671.51) | 106.82 (45.21-220.15） |
| Democratic Republic of the Congo | 3820184.82 (2956636.96-4844058.47） | 4677.81 (3635.61-5795.34） | 1118225.90 (833226.86-1451135.55） | 1282.99 (964.13-1658.13） | 5178.84 (1986.82-10533.98） | 3.90 (1.51-7.83） | 467321.23 (181637.64-948666.26) | 347.79 (137.97-700.08） |
| Libya | 21923.07 (16211.57-27786.54） | 276.40 (207.07-348.02） | 6333.27 (4688.32-8247.89） | 79.12 (58.56-102.82） | 8.50 (2.99-17.95） | 0.22 (0.08-0.46） | 749.69 (254.65-1562.02) | 19.18 (6.43-40.09） |
| Burundi | 232419.70 (179160.39-293238.45） | 2087.03 (1644.76-2585.44） | 72743.10 (55036.64-93741.63） | 609.59 (469.76-778.09） | 223.68 (90.10-454.69） | 1.14 (0.46- 2.37） | 19858.40 (8143.33-40635.07) | 94.43 (39.81-186.60） |
| Benin | 132178.11 (100788.27-168662.49） | 1089.92 (831.59-1361.36） | 39337.40 (28921.57-51236.09） | 309.26 (233.02-399.39） | 345.89 (133.63-680.68） | 1.47 (0.59-2.96） | 30974.93 (11870.82-61084.20) | 127.72 (51.10-248.28） |
| Monaco | 112.81 (87.30-143.53） | 400.32 (291.85-534.65） | 23.33 (18.08-29.09） | 77.61 (59.41-99.76） | 0.01 (0.01-0.02） | 0.06 (0.02-0.14） | 1.02 (0.44-2.08) | 5.80  (2.24-12.43） |
| Algeria | 127661.56 (95244.65-162543.05） | 279.00 (209.43-353.87） | 35959.20 (26729.27-47176.40） | 79.56 (59.02-104.00） | 127.95 (45.98-284.16） | 0.30 (0.11-0.65） | 11387.76 (4009.80-25440.44) | 26.00 (9.20-58.02） |
| Cambodia | 62480.93 (45714.86-80877.54） | 346.70 (259.45-441.46） | 19759.20 (14536.55-25690.42） | 107.70 (80.13-138.40） | 29.83 (11.55-61.66） | 0.18 (0.07-0.37） | 2666.28 (1031.52-5525.26) | 15.67 (6.13-32.32） |
| Azerbaijan | 24341.64 (18503.23-31060.62） | 205.67 (156.27-257.65） | 7053.83 (5344.19-9217.34） | 61.16 (45.63-79.33） | 15.65 (7.94-28.84） | 0.19 (0.09-0.38） | 1044.52 (497.80-2118.77) | 13.88 (6.17-28.54） |
| Croatia | 6007.67 (4651.06-7744.82） | 144.59 (108.00-186.67） | 1741.41 (1328.66-2254.50） | 44.96 (33.66-58.70） | 0.32 (0.22-0.50） | 0.00 (0.00-0.01） | 11.24 (8.18-16.13) | 0.19 (0.14-0.27） |
| C么te d'Ivoire | 460312.03 (349621.16-588783.10） | 1723.62 (1317.76-2193.20） | 122173.93 (89646.02-163427.55） | 441.55 (328.90-579.44） | 903.58 (352.81-1799.73） | 2.10 (0.86-4.21） | 80994.24 (31699.42-160429.47) | 183.32 (74.05-361.61） |
| Ghana | 621204.88 (478834.45-796879.84） | 1814.43 (1418.17-2255.69） | 168229.63 (125853.91-217316.84） | 473.23 (357.12-604.71） | 894.31 (316.30-1818.81） | 1.97 (0.71-3.96） | 80534.77 (28876.59-163541.69) | 175.53 (63.67-354.35） |
| Eswatini | 27050.05 (20913.94-35148.74） | 2277.33 (1771.87-2884.55） | 7481.51 (5521.30-9651.40） | 604.56 (455.94-773.63） | 40.15 (15.46-84.68） | 2.97 (1.18-6.18） | 3586.49 (1375.57-7581.76) | 261.49 (101.83-548.70） |
| Jordan | 43866.02 （33398.93-55808.42） | 331.96 （254.53-421.08） | 12390.36 （9084.96-16125.85） | 90.76 （67.00-117.05） | 44.06 （15.45-93.75） | 0.43 （0.15-0.90） | 3962.25 (1402.18-8414.73) | 38.14 （13.49-80.97） |
| Ethiopia | 2839776.28 （2190609.93-3698196.21） | 2841.20 （2205.26-3677.15） | 780273.34 （564852.18-1039004.40） | 733.96 （545.48-959.24） | 3125.73 （1161.05-6560.80 | 1.99 （0.77-4.10） | 281873.05 (106598.75-586749.25) | 174.60 （68.16-360.41） |
| Chad | 308264.42 (246353.66-386247.84） | 2073.83 (1631.57-2563.49） | 77355.78 (57775.48-100499.80） | 490.32 (368.85-630.79） | 993.10 （394.23-2043.57） | 2.61 （1.08-5.39） | 89303.68 (35534.74-184207.01) | 226.87 （95.18-457.83） |
| Ecuador | 160509.35 (123201.51-203692.81） | 855.23 (659.00-1076.32） | 45540.77 (34302.68-58912.44） | 239.84 （181.77-310.25） | 157.00 （56.73-328.92） | 1.01 （0.37-2.12） | 14039.89 (5093.14-29475.57) | 90.40 （32.67-189.80） |
| Afghanistan | 94960.81 （71104.69-123347.00) | 341.42 （256.84-428.08) | 26616.63 （19372.14-35196.10) | 87.89 64.94-115.25 | 336.95 (111.13-735.09) | 0.61 (0.22-1.38) | 30280.66 (10059.60-66094.63) | 53.18 (18.04-114.86) |
| Paraguay | 78297.99 (61118.98-97962.84) | 1028.25 (815.64-1269.77) | 18155.58 (13544.95-23681.96) | 234.00 (176.80-301.91) | 125.42 (47.60-268.31) | 2.02 (0.77-4.31) | 11141.61 (4131.38-23837.68) | 178.62 (66.09-382.46） |
| Lebanon | 18736.86 (13925.32-24128.53） | 307.57 (233.66-388.40） | 5202.65 (3859.74-6829.49） | 86.55 (64.13-113.84） | 11.78 (4.54-26.34） | 0.30 (0.11-0.67） | 1005.96 (366.01-2281.66) | 26.04 (9.31-59.47） |
| Equatorial Guinea | 75195.67 (57471.74-95030.12） | 4959.14 (3816.33-6196.37） | 22322.89 (16375.26-29220.43） | 1358.92 (1025.63-1773.72） | 74.55 (27.41-153.51） | 4.20 (1.57-8.62） | 6736.30 (2504.59-13809.77) | 376.91 (141.11-770.89） |
| Niue | 28.39 (20.67-39.00） | 1854.35 (1333.38-2574.16） | 4.07 (3.16-5.14） | 272.54 (208.92-347.47） | 0.02 (0.01-0.03） | 1.39 (0.49-2.93） | 1.48 (0.57-3.01) | 129.72 (48.09-266.79） |
| Kenya | 921023.34 (731934.27-1160049.99） | 1855.78 (1480.89-2323.00） | 240619.20 (178778.50-313954.77） | 470.43 (357.22-604.33） | 1102.67 (436.97-2245.15） | 1.99 (0.80-4.06） | 99801.36 (40011.20-200992.76) | 177.70 (72.41-355.30） |
| Kazakhstan | 37879.34 (28325.53-49030.17） | 193.64 (145.14-250.33） | 11290.09 (8404.23-14898.51) | 59.33 (44.11-78.48） | 2.79 (2.16-3.67） | 0.01 (0.01-0.02） | 242.96 (190.50-306.90) | 1.24 (0.97-1.56） |
| Hungary | 18711.98 (14874.74-22868.46 | 198.10 (153.97-243.60） | 4837.80 (3677.62-6144.04） | 55.38 (41.76-71.15） | 2.40 (1.72-3.35） | 0.01 (0.01-0.02） | 74.61 (56.71-99.30) | 0.50 (0.39-0.68） |
| Myanmar | 671382.49 (505242.15-856611.25） | 1149.8 (866.03-1471.31） | 168256.44 (125664.85-219541.62） | 284.14 (212.88-370.50） | 348.33 (129.77-726.45） | 0.68 (0.25-1.41） | 31416.07 (12002.53-65425.07) | 60.95 (23.21-127.02） |
| Malawi | 381902.65 (312852.45-454593.44） | 2256.58 (1864.22-2699.96） | 118512.06 (92891.95-146164.65） | 647.45 (508.80-789.68） | 369.19 (162.75-688.29） | 1.46 (0.67-2.76） | 33022.91 (14333.11-61077.20) | 125.35 (57.52-230.48） |
| San Marino | 106.64 (81.70-138.22） | 422.73 (302.96-584.43） | 21.40 (16.55-27.36） | 78.77 (59.86-100.73） | 0.01 (0.00-0.02） | 0.08 (0.03-0.18） | 0.91 (0.37-1.93) | 7.37 (2.59-16.34） |
| Sri Lanka | 43144.84 (32574.84-54972.04） | 188.54 (141.52-239.58） | 13818.64 (10245.59-17915.57） | 61.66 (45.74-79.90） | 14.84 (6.78-29.63） | 0.09 (0.04-0.19） | 1229.57 (551.37-2454.04) | 7.85 (3.13-16.20） |
| Uzbekistan | 71826.52 (53670.81-92181.54） | 197.28 (148.64-249.65） | 21617.33 (16069.26-28838.30） | 59.69 (44.57-79.23） | 3.79 (2.62-5.31） | 0.01 (0.01-0.02） | 347.75 (267.26-449.61) | 1.06 (0.81-1.37） |
| Serbia | 13831.36 (10477.17-17902.05） | 153.46 (114.65-194.82） | 3977.04 (2962.34-5234.58） | 46.07 (33.79-60.26） | 3.51 (1.59-7.07） | 0.09 (0.03-0.20） | 282.70 (118.57-591.82) | 7.91 (2.90-17.44） |
| Morocco | 486023.29 (361552.51-628173.29） | 1250.68 (932.91-1612.36） | 89567.46 (65282.94-120919.03） | 231.44 (168.92-313.28） | 723.26 (264.34-1553.38） | 2.35 (0.86-5.05） | 65009.82 (23736.97-139395.56) | 210.80 (76.91-452.27） |
| Mozambique | 1143745.69 (900081.95-1433041.20） | 4344.91 (3469.23-5343.73） | 310955.31 (232228.12-402760.70） | 1084.68 (825.54-1382.37） | 2291.57 (897.85-4851.38） | 4.51 (1.88-9.40） | 206128.03 (81375.80-436464.18) | 399.15 (160.76-834.47） |
| Guinea-Bissau | 30838.64 (23550.22-39903.36） | 1594.17 (1224.07-2019.87） | 8408.64 (6289.06-10953.19） | 413.44 (310.92-534.64） | 63.58 (25.75-133.60） | 2.03 (0.85-4.10） | 5670.72 (2289.02-11928.36) | 171.84 (71.34-356.20） |
